# The Rat Brain Transcriptome: From Infancy to Aging and Sporadic Alzheimer’s Disease-like Pathology

**DOI:** 10.3390/ijms24021462

**Published:** 2023-01-11

**Authors:** Natalia A. Stefanova, Nataliya G. Kolosova

**Affiliations:** Institute of Cytology and Genetics, Siberian Branch of Russian Academy of Sciences (ICG SB RAS), 10 Lavrentyeva Ave., Novosibirsk 630090, Russia

**Keywords:** Alzheimer’s disease, early postnatal period, brain, mitochondrial dysfunction, OXYS rat, RNA sequencing

## Abstract

It has been suggested that functional traits of the adult brain—all of which are established early in life—may affect the brain’s susceptibility to Alzheimer’s disease (AD). Results of our previous studies on senescence-accelerated OXYS rats, a model of sporadic AD, support this hypothesis. Here, to elucidate the molecular genetic nature of the aberrations revealed during brain maturation, we analyzed transcriptomes (RNA-seq data) of the prefrontal cortex (PFC) and hippocampus of OXYS rats and Wistar (control) rats in the period of brain maturation critical for OXYS rats (ages P3 and P10; P: postnatal day). We found more than 1000 differentially expressed genes in both brain structures; functional analysis indicated reduced efficiency of the formation of neuronal contacts, presumably explained mainly by deficits of mitochondrial functions. Next, we compared differentially expressed genes in the rat PFC and hippocampus from infancy to the progressive stage of AD-like pathology (five ages in total). Three genes (*Thoc3*, *Exosc8*, and *Smpd4*) showed overexpression in both brain regions of OXYS rats throughout the lifespan. Thus, reduced efficiency of the formation of neural networks in the brain of OXYS rats in infancy likely contributes to the development of their AD-like pathology.

## 1. Introduction

The notion that the preclinical period of sporadic Alzheimer’s disease (AD), the most common late-life dementia, can last for decades is supported by studies involving MRI and positron emission tomography (PET), which indicate that there are synergistic relations between tau protein deposition, amyloid β peptide (Aβ) plaques, and neurodegeneration [1,2,3,4]. Emerging evidence suggests that Aβ may be necessary but not sufficient as a cause of AD, and Aβ’s major pathological effects, many of them tau-dependent, culminate years later in neurodegeneration [5,6].

An alternative explanation of the disease mechanism is linked with mitochondrial dysfunction, which has been well documented for AD [7]. Age-associated loss of mitochondrial function affects the expression and processing of AβPP, which initiate Aβ accumulation [8]. In contrast to normal aging, human hippocampal AD signatures feature predominately downregulated expression of genes associated with neuronal and mitochondrial processes [9]. It has been suggested that mitochondrial dysfunction early in life may play a decisive role in the development of AD later in life. This idea is supported by studies on multiple transgenic mouse models of familial AD, indicating abnormal mitochondrial axonal trafficking observed already in the embryonic period, with additional abnormalities in fission, fusion, and function detected prior to the development of amyloid plaques or memory impairment [10]. Nonetheless, transgenic mouse models of AD do not appear to consistently simulate the strong influence of transcriptional aberrations observed in human AD, although transcriptional profiles of brain aging are concordant between rodents and humans [9].

The hypothesis that early life factors, primarily genetic and environmental factors that cause atypical brain development, cause the adult brain to become more susceptible to a neurodegenerative pathology in old age deserves more attention [11,12,13,14]. Epidemiological studies have shown [15] that a possible factor determining future trends of brain development and the risk of AD is low weight at birth, which in turn may be caused by preterm delivery as well as trophic insufficiency during gestation. Recent evidence points to brain alterations associated with late preterm birth, and emerging evidence suggests that these alterations are associated with poorer neurodevelopment in early childhood [16]. Moreover, in a recent cohort study, it was demonstrated that lower early life cognitive abilities are associated with higher odds of AD later in life [17]. It has been proposed that anatomical traits (quantity and connectivity of neurons) and functional traits (an ability to engage alternative brain networks) of the adult brain—all of which are established early in life—may influence brain susceptibility to AD and the brain’s ability to use compensatory reserves [18].

The results of our work on senescence-accelerated OXYS rats, a model of sporadic AD, which spontaneously develop all the major signs of AD and largely reproduce the stages of the disease [19,20], have confirmed the validity of this hypothesis. In OXYS rats in the early postnatal period, we have identified the features of brain maturation that can act as prerequisites for the development of initial neurodegenerative changes at a later age. OXYS rats show decreased duration of gestation (compared with their maternal Wistar rat strain), a lower body weight at birth, delayed physical development and delayed formation of reflexes in the postnatal period against the background of impaired neuronal stem cells differentiation and the formation of mossy fibers in the dentate gyrus of the hippocampus [21,22]. Aβ accumulation, tau protein hyperphosphorylation, and the cognitive decline later in life are preceded by lack of glial support and by mitochondrial hypofunction [20,23].

Here, to elucidate the nature of the revealed changes in brain maturation, we analyzed transcriptomes of the prefrontal cortex (PFC) and hippocampus of OXYS and Wistar rats at ages P3 and P10 (P: postnatal day), which according to our estimates are the critical time points of brain maturation in OXYS rats. In addition, we performed a comparative gene expression analysis in the rat PFC and hippocampus from infancy to aging and to AD-like pathology using current and previous RNA sequencing (RNA-seq) data [24,25,26] as a comprehensive approach to understanding the molecular genetic mechanisms of development, aging, and dementia.

## 2. Results

### 2.1. Differential Expression of Genes in the PFC and Hippocampus of OXYS Rats in the Early Postnatal Period

**Figure 1:**
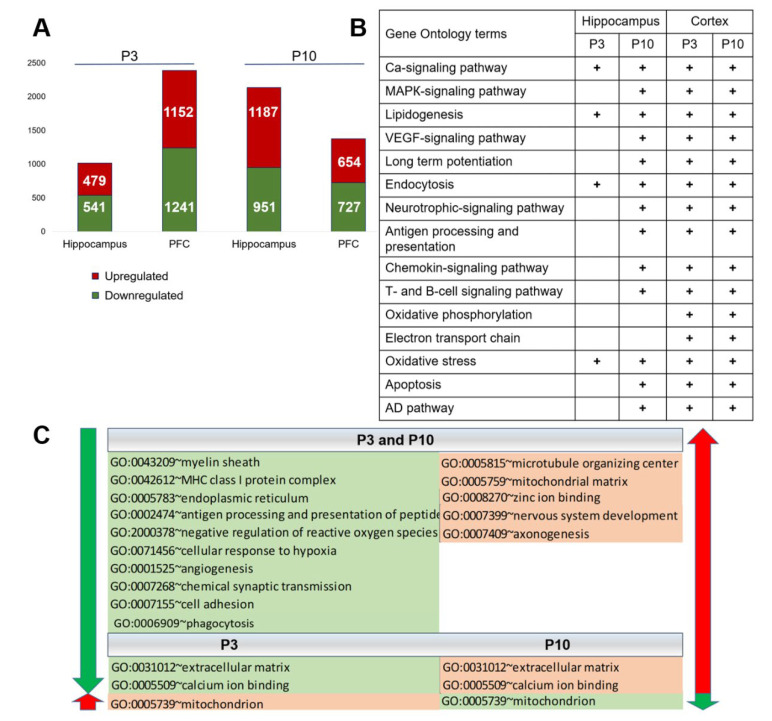
Differential expression of genes: the effect of the genotype (strain). (**A**) The number of DEGs in the PFC and hippocampus of 3- and 10-day-old OXYS rats compared to age-matched Wistar rats. The numbers of DEGs are marked red if upregulated and green if downregulated. (**B**) Pathways (according to KEGG and Wiki pathways) that undergo changes in OXYS rats, as compared to age-matched Wistar rats. (**C**) The most significant GO terms in the PFC and hippocampus of OXYS rats according to DAVID are highlighted in red if upregulated and in green if downregulated.

To determine gene expression changes associated with the delayed brain maturation in OXYS rats, we performed RNA-seq analysis in the PFC and hippocampal tissue from 3- and 10-day-old OXYS and Wistar rats (as a control strain). According to the RNA-seq results, at age P3, 2393 genes in the PFC and 1020 in the hippocampus of OXYS rats are differentially expressed as compared to Wistar rats (P_adj_ < 0.05; Figure 1A). At age P10, there were 1,381 differentially expressed genes (DEGs) in the PFC and 2138 DEGs in the hippocampus of OXYS rats (P_adj_ < 0.05; Figure 1A). Analysis of the 100 top DEGs (Tables S1–S4) using DAVID revealed basic clusters that at P3 were related to “extracellular matrix organization” in the PFC and to “cilium assembly” in the hippocampus (Table S5). The primary cilium is an antenna-like organelle that is essential for interpreting extracellular signals that regulate growth, development, and homeostasis. At P10, basic clusters of Gene Ontology (GO) terms were related to “oxygen processes”, “focal adhesion”, and “antigen processing and presentation” in the PFC and to “mitochondrial respiratory chain complex I” and “endoplasmic reticulum” in the hippocampus (Table S5).

According to analyses of KEGG and Wiki pathways (*p* < 0.01; Figure 1B), at both ages and in both brain regions of OXYS rats, changes in gene expression are associated with the Ca^2+^ signaling pathway, lipid metabolic process, endocytosis, and oxidative stress. It should be noted that prevalence levels of signaling pathways and processes in the PFC at P3 and P10 and in the hippocampus at P10 are identical, despite differences in the number of DEGs (Figure 1A). These genes are associated with MAPK, VEGF, neurotrophic, chemokine, T-cell, and B-cell signaling pathways, antigen processing and presentation, long-term potentiation, and apoptosis (Figure 1B). It is noteworthy that already at these ages, changes in gene expression in the brain of OXYS rats are associated with the AD pathway (Figure 1B), namely with processes related to processing and degradation of Aβ, microtubule-associated protein tau, synapses, and mitochondrial respiratory chain complex (Figures S1–S3).

As for the direction of changes in gene expression, both in the PFC and in the hippocampus at both ages, reduced gene expression is associated with myelination, immune processes, synaptic transmission, and angiogenesis, whereas increased gene expression is associated with microtubule organization, axonogenesis, and development of the nervous system (Figure 1C). As for the mitochondria, the increased gene expression at P3 and reduced gene expression at P10 are associated with the GO term “mitochondrion” (Figure 1C).

**Figure 2:**
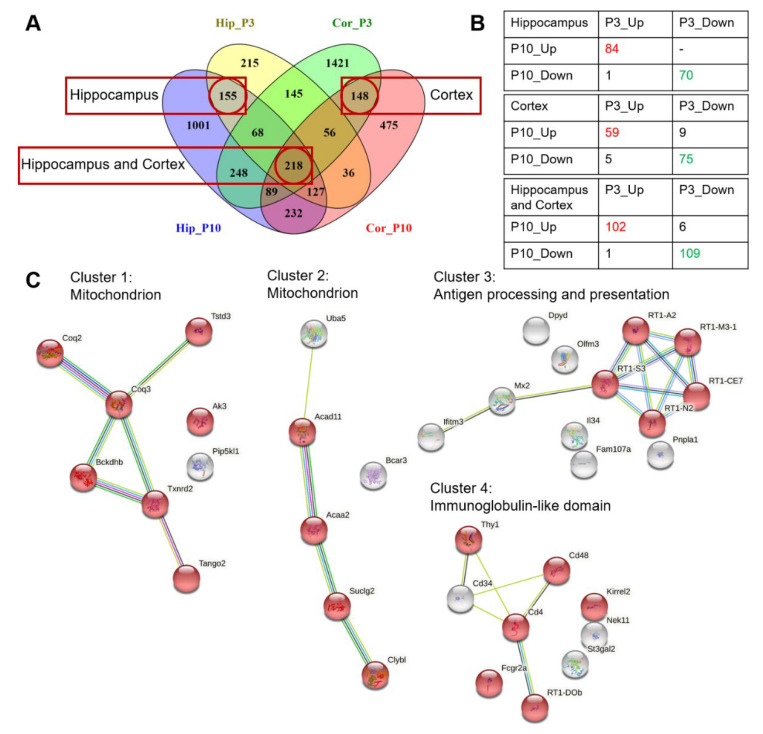
Differential expression of genes: the effect of the genotype. (**A**) The matching DEGs in the PFC and hippocampus of 3- and 10-day-old OXYS rats compared to age-matched Wistar rats. (**B**) The numbers of up- and downregulated common DEGs in the PFC and hippocampus of OXYS rats. (**C**) The most significant clusters in OXYS rats according to STRING for downregulated DEGs that matched among the two age periods and two brain regions.

Next, analysis of the 155 matching DEGs in the hippocampus (between the two age-specific overlapping gene sets; Figure 2A) using STRING revealed no significant enrichment within the set of 84 upregulated DEGs, and for the set of 70 downregulated DEGs, significantly enriched GO terms are related to synaptic processes (Figure 2B; Table S6). Among the matching 148 DEGs in the PFC (the two age-specific overlapping gene sets; Figure 2A), in the set of 59 upregulated DEGs, no significant enrichment was detected, and for the set of 75 downregulated DEGs, significantly enriched GO terms are related to anatomical structure development and phosphoprotein (Figure 2B; Table S6). Among the 218 DEGs that matched among the two age periods and two brain structures (the overlap of gene sets; Figure 2A), mRNA expression of almost all of them changed in the same direction in OXYS rats (102 upregulated and 109 downregulated DEGs; Figure 2B). In the set of upregulated DEGs, no significant enrichment was detected, and for the set of downregulated DEGs, the most significantly enriched GO terms (Table S6) and clusters (Figure 2C) are associated with mitochondrial and immune processes.

### 2.2. Genes Differentially Expressed between OXYS and Wistar Rats Related to Aβ Function in the Early Postnatal Period

**Figure 3:**
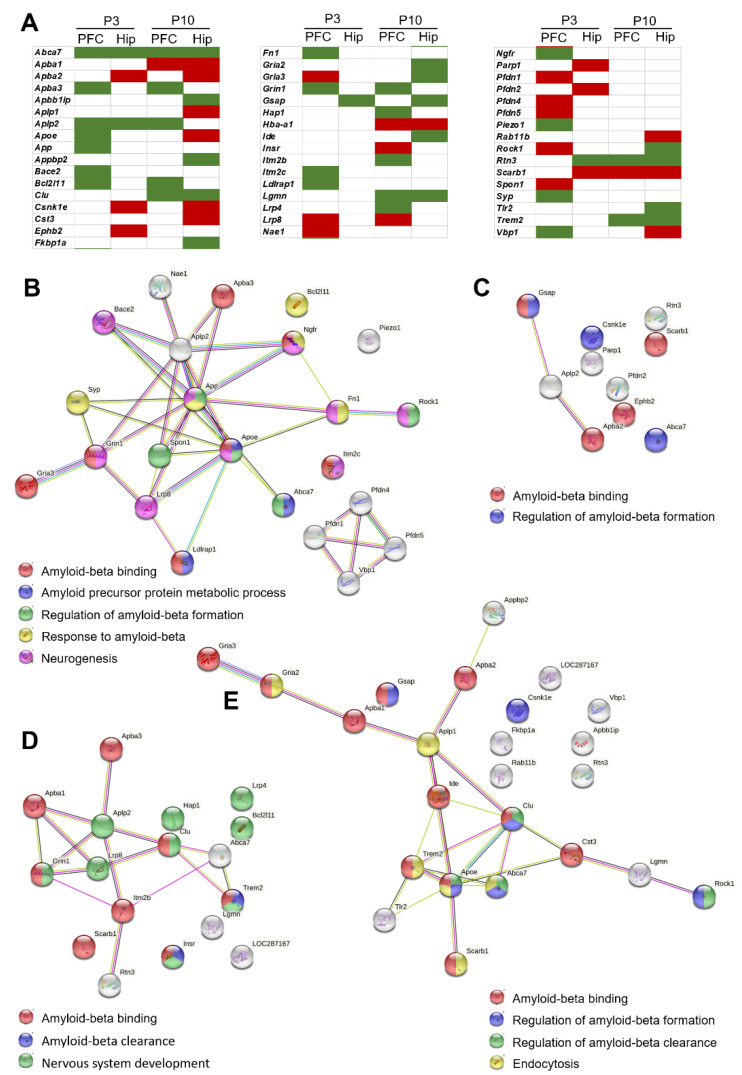
Changes in gene expression related to the function of Aβ in OXYS rats. (**A**) Up- and downregulated DEGs in the PFC and hippocampus of 3- and 10-day-old OXYS rats compared to age-matched Wistar rats. GO terms according to STRING for sets of DEGs (**B**) in the PFC at P3, (**C**) in the hippocampus at P3, (**D**) in the PFC at P10, and (**E**) in the hippocampus at P10.

Here, we found that already in the early postnatal period, OXYS rats showed changes in gene expression related to the function of Aβ (AD pathway; Figures S1–S3), which on the one hand, appears to be a necessary cause of AD [4], and on the other hand, plays an important role during brain development. We next performed analysis of transcriptomic changes associated with the function of Aβ, using a list of 118 genes from the Rat Genome Database (RGD). In the hippocampus of OXYS rats at P3, we identified 23 DEGs in the PFC and 10 DEGs related to Aβ processes. There were 17 DEGs in the PFC and 24 DEGs in the hippocampus of OXYS rats at P10 (Figure 3A). Among them, the expression of gene *Abca7* (ATP-binding cassette subfamily A member 7), which is now considered an important genetic determinant of late-onset AD [27], was lower in OXYS rats in both brain regions and at both ages (log_2_fold change = −0.56; P_adj_ = 6.53 × 10^−5^ for PFC at P3; log_2_fold change = −0.39, P_adj_ = 2.33 × 10^−4^ for hippocampus at P3; log_2_fold change = −0.52, P_adj_ = 9.07 × 10^−4^ for PFC at P10; log_2_fold change = −0.21, P_adj_ = 2.11 × 10^−3^ for hippocampus at P10). Three genes underwent unidirectional expression changes in both the PFC and hippocampus at one or two ages. The expression of *Aplp2* (amyloid beta precursor-like protein 2) was lower at P3 in both brain regions and at P10 in the PFC of OXYS rats. Although APLP2 lacks the Aβ region, its processing by the same secretases is similar to that of APP, and APLP2 has important physiological functions during brain development and in neuronal plasticity, memory, and neuroprotection in the mature and ageing brain [28]. The expression of gene *Rtn3* (reticulon 3), whose product inhibits BACE1 activity and APP processing, was lower at P3 in the hippocampus and at P10 in both brain regions. The *Scarb1* gene (scavenger receptor class B member 1), which is a microglial Aβ receptor, was upregulated at P3 in both brain regions and at P10 in the hippocampus. The expression of some genes was age-dependent or brain region-dependent, and all of these DEGs underwent unidirectional changes. The expression of five genes changed at P10 in both the PFC and hippocampus (upregulated: *Apba1* and *Hba-a1*; downregulated: *Clu*, *Trem2*, and *Lgmn*). The *Clu* gene (clusterin), *Trem2* (triggering receptor expressed on myeloid cells 2), and *Abca7* are related to microglial function [29], and downregulation of these genes may be indirectly related to the microglial hypofunction detected recently in OXYS rats in the early postnatal period [23]. Because microglia are heavily implicated in the shaping of the synaptic landscape of the brain during the incorporation of new memories and experiences into the neural network, the CNS immune system is also thought to be involved in cognitive function [30]. In addition, the expression of four genes changed at P3 and P10 exclusively in the PFC (upregulated: *Lrp8*; downregulated: *Apba3*, *Bcl2l11,* and *Grin1*). The expression of three genes changed at P3 and P10 exclusively in the hippocampus (upregulated: *Apba2* and *Gsnk1e*; downregulated: *Gsap*).

According to functional analysis, at P3, DEGs in the PFC were related to GO terms “Aβ binding”, “amyloid precursor protein metabolic process”, “regulation of Aβ formation”, “response to Aβ”, and “neurogenesis” (Figure 3B); DEGs in the hippocampus were related to “Aβ binding” and “regulation of Aβ formation” (Figure 3C). At P10, DEGs in the PFC were related to GO terms “Aβ binding” and “Aβ clearance”; DEGs in the hippocampus were associated with “Aβ binding”, “regulation of Aβ formation”, “regulation of Aβ clearance”, and “endocytosis” (Figure 3E).

### 2.3. Genes—Differentially Expressed between OXYS and Wistar Rats—Related to Mitochondrial Function in the Early Postnatal Period

**Figure 4:**
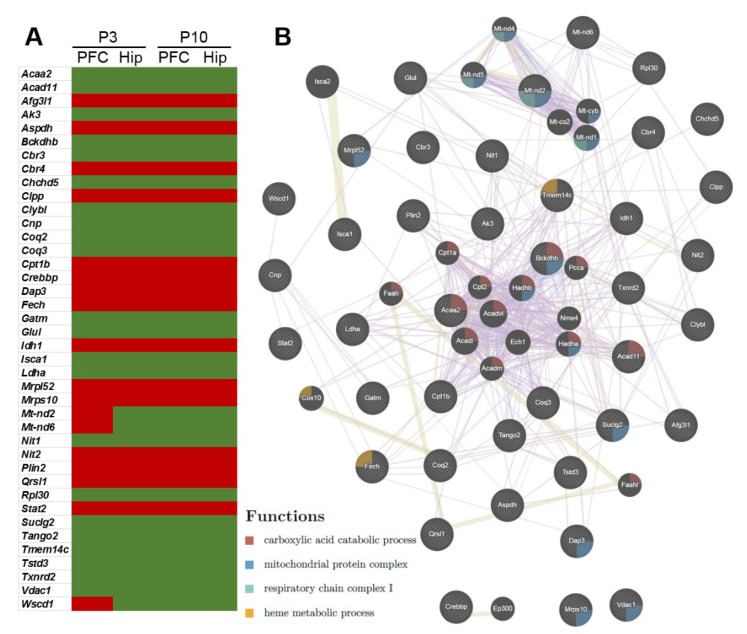
Changes in expression of matching genes related to mitochondrial function in the PFC and hippocampus of 3- and 10-day-old OXYS rats compared to age-matched Wistar rats. (**A**) Up- and downregulated matching DEGs in OXYS rats. (**B**) GO terms that (according to GeneMania) are enriched within the set of 40 DEGs that matched among the two age periods and two brain regions.

We next performed a more complete analysis of transcriptomic changes associated with the functional category of mitochondria at the early postnatal period in OXYS rats, using a combined list of 2080 genes from three databases (MitoCarta 2.0, IMPI, and RGD) [25]. We found that in OXYS rats, at P3 in the PFC, 10.5% of DEGs (250 genes), and in the hippocampus, 13% of DEGs (134 genes), whereas at P10 in the PFC, 11.5% of DEGs (160 genes), and in the hippocampus, 12% of DEGs (255 genes) were associated with mitochondrial function, confirming the hypothesis that the delayed brain maturation in OXYS rats is strongly associated with mitochondrial abnormalities. Among them, 40 DEGs matched among the two age points and two brain regions in OXYS rats. Almost all of these DEGs showed unidirectional changes in gene expression: mRNA expression of 15 genes increased and that of 22 genes decreased in the PFC and hippocampus at P3 and P10 (Figure 4A). Analysis of the 40 matching DEGs (Figure 4B) using GeneMania revealed four functional processes that were related to carboxylic acid catabolic process (false discovery rate [FDR] = 1.75 × 10^−10^; the expression of genes *Acaa2*, *Acad11,* and *Bckdhb* was downregulated), mitochondrial protein complex (FDR = 1.25 × 10^−9^; the expression of genes *Dap3*, *Mrpl52,* and *Mrps10* was upregulated; the expression of genes *Bckdhb*, *Suclg2,* and *Vdac1* was downregulated), respiratory chain complex I (FDR = 8.25 × 10^−3^; the expression of gene *Mt-nd2* was upregulated in the cortex at P3 and downregulated in the hippocampus at P3 and in both brain regions at P10), and heme metabolic process (FDR = 1.75 × 10^−2^; the expression of gene *Fech* was upregulated, and the expression of gene *Tmem14c* was downregulated).

According to the functional annotation clustering with DAVID, the most significant five clusters at P3 in the PFC are related to oxidative phosphorylation (enrichment score [ES] = 10.14), mitochondrial respiratory chain complex IV (ES = 4.65), response to xenobiotic stimulus (ES = 4.03), mitochondrial proton-transporting ATP synthase complex (ES = 3.84), and electron transport and mitochondrial respiratory chain complex I (ES = 3.80). In the hippocampus at this age, the clusters are related to lipid metabolism (ES = 4.96), oxidative phosphorylation, and mitochondrial respiratory chain complex I (ES = 4.89), tricarboxylic acid cycle (ES = 4.82), acyl-CoA dehydrogenase activity (ES = 2.93), and glycolysis/gluconeogenesis (ES = 2.88) (Table S7). At P10 in the PFC, the most significantly enriched five clusters are related to oxidative phosphorylation (ES = 9.62), lipid metabolism (ES = 6.79), fatty acid beta-oxidation and acetyl-CoA C-acetyltransferase activity (ES = 4.93), mitochondrial intermembrane and mitochondrial respiratory chain complex IV (ES = 3.31), and peroxisome (ES = 2.59). In the hippocampus at this age, the clusters are related to oxidative phosphorylation (ES = 13.13), lipid metabolism (ES = 5.70), mitochondrial respiratory chain complex IV (ES = 5.03), proton-transporting ATP synthase activity, rotational mechanism (ES = 4.12), and peroxisome (ES = 3.72) (Table S7).

**Figure 5:**
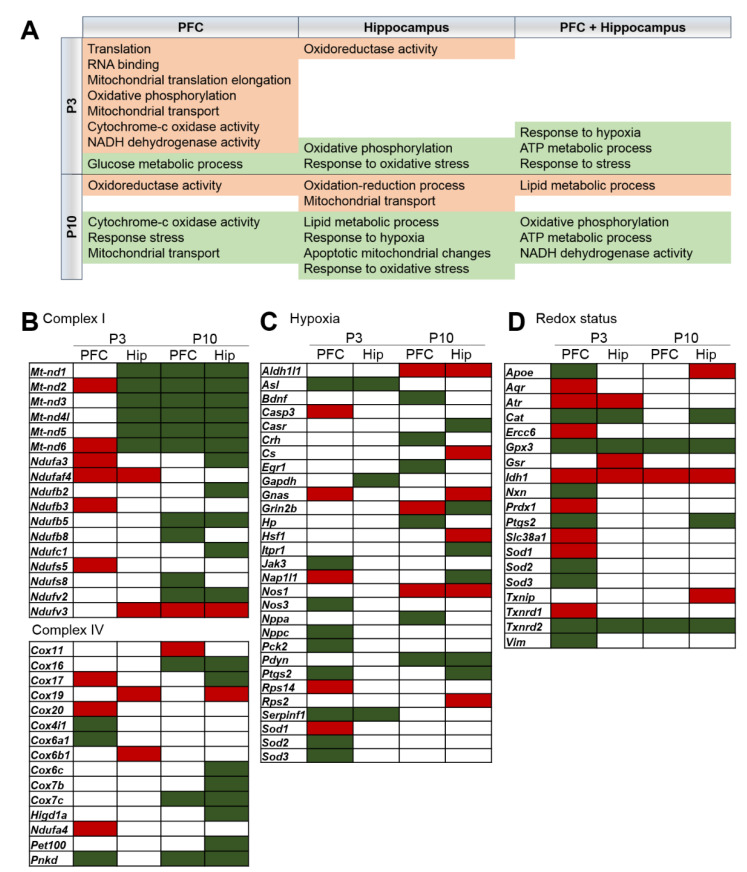
Genes—differentially expressed between OXYS and Wistar rats—related to mitochondrial function in the PFC and hippocampus at P3 and P10. (**A**) GO terms in the PFC and hippocampus of OXYS rats according to cluster analysis by STRING are marked red if upregulated and green if downregulated. In OXYS rats, up- and downregulated DEGs related to (**B**) mitochondrial respiratory chain complex I and IV, (**C**) hypoxia, and (**D**) oxidative stress and antioxidant defense.

As for the direction of changes in gene expression, according to the cluster analysis by STRING, increased gene expression at P3 in the PFC is associated with translation, RNA binding, mitochondrial translation elongation, oxidative phosphorylation, mitochondrial transport, cytochrome-c oxidase activity, and NADH dehydrogenase (ubiquinone) activity (Figure 5A and Figure S4); in the hippocampus, it is associated with oxidoreductase activity (Figure 5A and Figure S5). At P10, the increased gene expression in both brain regions is associated with oxidoreductase activity and lipid metabolic process, and in the hippocampus, in addition, it is associated with mitochondrial transport (Figure 5A, Figures S6 and S7). It is noteworthy that reduced gene expression is associated mainly with the same processes: ATP metabolic process, oxidative phosphorylation, NADH dehydrogenase activity, cytochrome-c oxidase activity, response to hypoxia, response to stress, and response to oxidative stress (Figure 5A and Figures S4–S7).

Given that changes in the expression of genes were associated with GO terms “mitochondrial respiratory chain complex”, “response to hypoxia”, and “response to oxidative stress”, we performed analyses of genes related to mitochondrial respiratory chain complexes I-V (PathCards) and hypoxia (RGD) and genes involved in oxidative stress and antioxidant defense [31]. For mitochondrial respiratory chain complexes, the most significant changes in gene expression were related to complexes I and IV (Figure 5B), especially to genes coding mitochondrial DNA (mtDNA) complex I: *Mt-nd1*, *Mt-nd2*, *Mt-nd3*, *Mt-nd4l*, *Mt-nd5*, and *Mt-nd6*. Their expression was downregulated in the hippocampus at P3 and P10 and in the PFC at P10. As for hypoxia, oxidative stress and antioxidant defense, we found no clear evidence pointing to hypoxia or oxidative stress in the brain of OXYS rats. Expression of only three genes (*Gpx3*, *Idh1*, and *Txnrd2*) that participate in redox-related processes changed in both brain regions and at both ages in OXYS rats. The expression of isocitrate dehydrogenases (*Idh1*), which plays a critical role in the generation of NADPH, proved to be upregulated. The expression of thioredoxin reductase 2 (*Txnrd2*), which plays a key role in redox homoeostasis, was downregulated. Metabolic control of oxidation and reduction reactions within the cell is maintained in part by key enzymes including superoxide dismutase, catalase, and glutathione peroxidase and reductase [32]. Among these enzymes, glutathione peroxidase 3 (*Gpx3*) was downregulated; glutathione reductase (*Gsr*) and superoxide dismutase 1 (*Sod1*) turned out to be upregulated, and *Sod2* and *Sod3* were underexpressed in the PFC at P3. The expression of catalase (*Cat*) was lower in OXYS rats in the hippocampus at both ages and in the PFC at P3.

### 2.4. Similarities and Differences in Gene Expression in the PFC and Hippocampus between OXYS and Wistar Rats during the Early Postnatal Period

**Figure 6:**
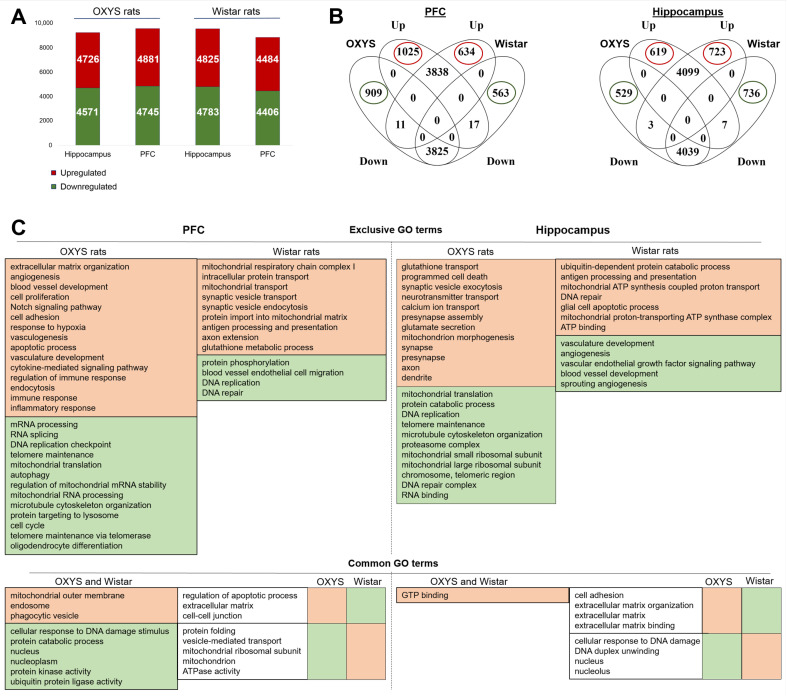
Differential expression of genes: the effect of age. (**A**) The numbers of genes that significantly changed expression in the PFC and hippocampus between ages P3 and P10 in OXYS and Wistar rats. The numbers of DEGs are red if upregulated and green if downregulated. (**B**) The numbers of DEGs either matching or exclusive for OXYS and Wistar rats (in the PFC and hippocampus). (**C**) The most significantly enriched GO terms that are exclusive, common, or common but with opposite directions of changes between OXYS and Wistar rats in the PFC and hippocampus, according to DAVID. The GO terms are red if upregulated and green if downregulated.

We found that in OXYS rats, 9626 genes significantly changed expression in the PFC and 9297 genes changed expression in the hippocampus between ages P3 and P10. In Wistar rats, 8890 genes significantly changed expression in the PFC and 9608 genes changed expression levels in the hippocampus between the ages P3 and P10 (Figure 6A).

To determine the functional meaning of the gene expression changes that occur during normal brain maturation in Wistar rats and specific features (that can act as prerequisites for the development of initial neurodegenerative changes at a later age) of brain maturation in OXYS rats in the early postnatal period, we performed KEGG and Wiki pathway analyses at *p* < 0.01. We found that by age P10, annotated pathways in the PFC and hippocampus in almost all cases were similar between the two rat strains. Between the top 50 significant pathways in the PFC and hippocampus (P_adj_ < 1.45 × 10^−17^ and P_adj_ < 4.71 × 10^−16^, respectively) identified in OXYS rats and the top 50 significant pathways (P_adj_ < 5.25 × 10^−15^ and P_adj_ < 2.62 × 10^−19^, respectively) identified in Wistar rats (Tables S8–S11), the matching pathways are related to synaptic and immune processes, the cell cycle, focal adhesion, and RNA processes.

To determine which processes are similar or different and play more important roles (i.e., are enriched) in OXYS and in Wistar rats between the two age points, we next compared the GO terms for sets of exclusively up- or downregulated DEGs in each rat strain. In the PFC of OXYS rats, between the ages of P3 and P10, the set of 1025 genes exclusively showing upregulated expression (Figure 6B) was found to be mainly enriched with vascular and immune processes (Figure 6C). The set of 909 genes exclusively downregulated in OXYS rats proved to be enriched with RNA processing, telomere maintenance, and mitochondrial RNA processing. In the PFC of Wistar rats during the same period, there were 634 genes that showed exclusively upregulated expression (Figure 6B); this gene set was mainly enriched with mitochondrial, synaptic, and immune processes (Figure 6C). The set of 563 exclusively downregulated genes was related to DNA replication and repair, protein phosphorylation and blood vessel endothelial cell migration. In addition, in the PFC, the common GO terms with opposite directions of gene expression between OXYS and Wistar rats are presented in Figure 6C. In particular, the DEGs related to the extracellular matrix, cell–cell junction, and regulation of apoptotic process were upregulated in OXYS rats and downregulated in Wistar rats. The DEGs related to the mitochondrion, ATPase activity, protein folding, and vesicle-mediated transport were downregulated in OXYS rats and upregulated in Wistar rats (Figure 6C). Upregulated and downregulated GO terms common between the two rat strains in the PFC are presented in Figure 6C.

In the hippocampus of OXYS rats, between ages P3 and P10, there were 619 genes that were exclusively upregulated and 529 genes that were exclusively downregulated (Figure 6B). The upregulated DEGs were mainly related to synaptic processes; the downregulated DEGs were related to mitochondrial function, DNA and RNA processes, and telomere maintenance (Figure 6C). In Wistar rats during the same period, there were 723 and 736 genes that were exclusively up- and downregulated, respectively (Figure 6B). The upregulated DEGs were mainly related to immune and mitochondrial processes; the downregulated DEGs were related to vascular processes (Figure 6C). The analysis of the common GO terms with opposite directions of gene expression between OXYS and Wistar rats in the hippocampus showed that the processes related to the extracellular matrix were upregulated in OXYS rats and downregulated in Wistar rats, and the processes related to DNA and nucleus were downregulated in OXYS rats but upregulated in Wistar rats. It is notable that in OXYS rats, the only common GO term between up- and downregulated DEGs was “mitochondrion”. The only common GO term between the two rat strains was GTP binding, which was upregulated.

Next, analysis of 231 matching exclusive DEGs in the PFC and hippocampus (between overlapping gene sets of the two brain structures) of OXYS rats using STRING revealed that in the set of 115 upregulated DEGs, the significantly enriched GO term is extracellular matrix (*p* < 0.03), and in the set of 116 downregulated DEGs, the significantly enriched GO terms are RNA metabolic process and mitochondrial processes. In Wistar rats, among 150 DEGs matching exclusive for the two brain structures, 76 upregulated DEGs are related to mitochondrial processes, and in the set of 74 downregulated DEGs, no significant enrichment was detected.

### 2.5. Comparative Gene Expression Analysis in the PFC and Hippocampus of OXYS Rats in the Period Preceding the Development of AD signs and during Their Manifestation and Active Progression

**Figure 7:**
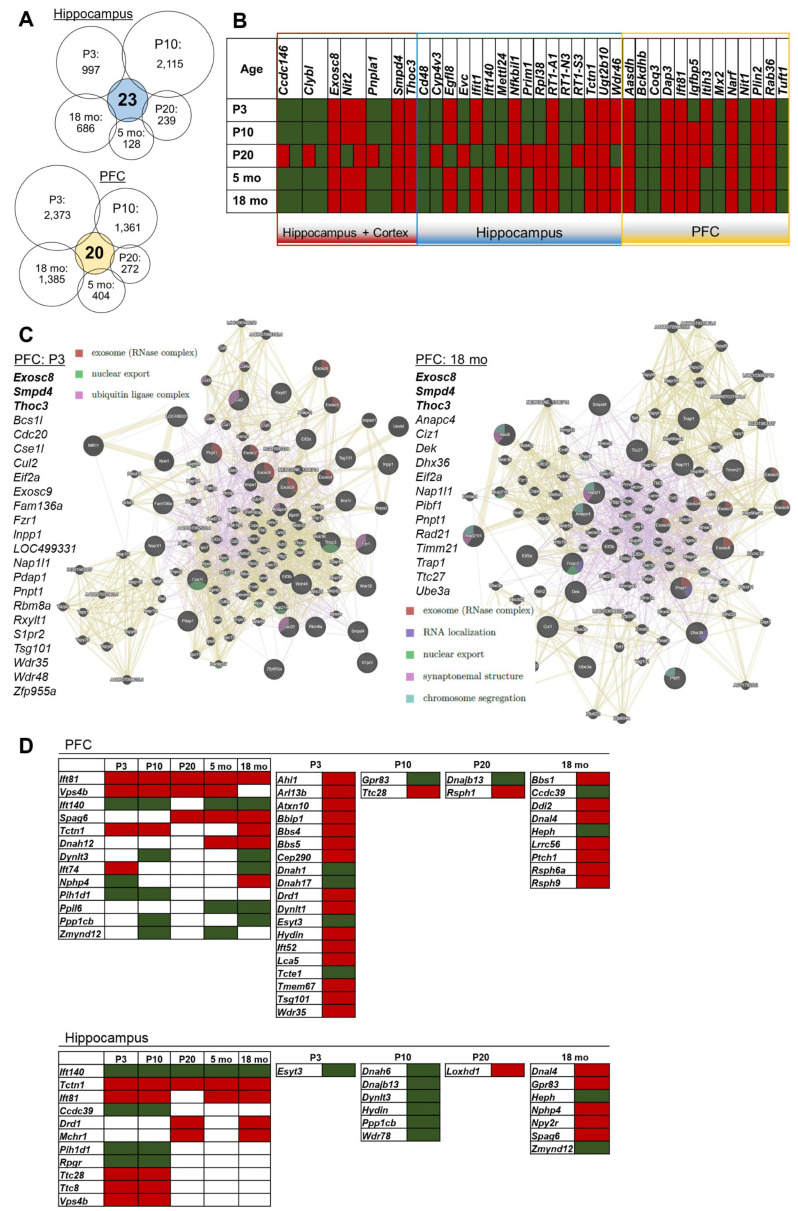
Comparative gene expression analysis in the PFC and hippocampus of OXYS rats at ages P3, P10, P20, 5 months, and 18 months. (**A**) The numbers of DEGs common among ages P3 to 18 months in the PFC and hippocampus of OXYS rats. (**B**) The matching genes that significantly changed expression in the PFC and hippocampus between ages P3 and 18 months in OXYS rats. The DEGs are red if upregulated and green if downregulated. (**C**) In the PFC of OXYS rats at P3 and 18 months, DEGs related to *Smpd4*, *Thoc3*, and *Exosc8* according to GeneMania. (**D**) DEGs related to cilia in the PFC and hippocampus of OXYS rats are marked red if upregulated and green if downregulated.

Next, to identify possible AD-related candidate genes, we performed a comparative analysis of DEGs in the PFC and hippocampus of OXYS rats, starting from P3 and ending at 18 months (a period of pronounced neurodegenerative changes) using current and previous RNA-seq data [24,25,26]. In the PFC, 20 common genes were identified (Figure 7A); functionally they are annotated with mitochondrial functions and lipid processes. There are 23 DEGs in the hippocampus (Figure 7A), and they are associated with antigen processing and presentation of the major histocompatibility complex. The expression of some of them changed in both brain structures, while the rest changed expression only in the hippocampus or cortex (Figure 7B). It should be noted that the directions of their expression change were the same, which was either reduced throughout the lifespan or increased. Nonetheless, at P20 (the period of completion of brain maturation) and only in the hippocampus, the direction of expression change of half of the genes differed from that at all other ages.

In both brain structures, the expression of three genes changed unidirectionally (was upregulated) throughout the lifespan: *Smpd4*, sphingomyelin phosphodiesterase 4; *Thoc3*, THO complex 3; and *Exosc8*, exosome component 8. The protein encoded by *Smpd4* is a sphingomyelinase that catalyzes the hydrolysis of membrane sphingomyelin to produce phosphorylcholine and ceramide. This gene is activated by DNA damage, cellular stress, and tumor necrosis factor [33]. The *Thoc3* gene codes for a component of the nuclear THO transcription elongation complex, which is a part of the larger transcription export (TREX) complex, which couples messenger RNA processing and export [34]. The *Exosc8* gene encodes the 3’-5’ exoribonuclease that specifically interacts with mRNAs containing AU-rich elements. The encoded protein is a component of the exosome complex that is important for the degradation of numerous RNA species [35]. When we looked at the relations among these three genes, it turned out that they are co-expressed. Functionally, the most significant GO annotations are associated with RNA. Next, we looked at whether the expression of genes from this gene network (103 genes total) changed in the PFC and hippocampus at different ages in OXYS rats. According to the results of the analysis, in OXYS rats, the largest number of the DEGs from this network was present in the hippocampus at P10 (16 genes). In the PFC at our extreme points [P3 (23 genes) and 18 months (16 genes)], changes in gene expression functionally overlapped very strongly and were associated primarily with the RNA exosome (Figure 7C).

In addition, the expression changes of *Ift140* in the PFC and of *Tctn1* and *Ift81* in the hippocampus were unidirectional in OXYS rats. These genes are related to cilia: the dynamic microtubule-based organelles that play an important role in brain development; cilia defects are implicated in a growing list of human disorders [36]. Considering that analysis of 100 top DEGs in the hippocampus of OXYS rats at P3 identified the most significant cluster associated with cilia (Table S5), we decided to analyze the changes in gene expression related to cilia at all ages in more detail. We used a list of 165 cilia-related genes [36], which includes intraflagellar transport complexes’ proteins, transition zone proteins, ciliary membrane proteins, and proteins restricted to motile cilia. Among them, the largest number of the DEGs from the cilia gene list were present in the PFC at P3 (26 genes), at P10 (10 genes), and at 18 months (19 genes) and in the hippocampus at P3 (11 genes), at P10 (15 genes), and at 18 months (12 genes) (Figure 7D). These data were suggestive of an important role of cilia in the delayed brain development and progression of AD-like pathology in OXYS rats.

## 3. Discussion

To answer the main question, changes in which signaling pathways and processes become the most pronounced in the delayed maturation of the brain in OXYS rats [22,23,37], and to determine their potential role in the development of AD signs later in life [20], here we evaluated the transcriptomic changes in the PFC and hippocampus of OXYS and Wistar rats at the earliest stages of their postnatal life. It is believed that the level of brain development of rat pups at P1–P3 is similar to the level of human brain development at 23–32 weeks of gestation, and the level of brain development of rat pups at P7–P10 is equivalent to 36–40 weeks of gestation or the child brain at birth [38]. Long-standing anatomic data and recent imaging data indicate that cerebral cortical neuronal maturation in humans is especially exuberant during the last weeks of gestation [39]. Therefore, this rapidity and complexity render the cerebral cortex vulnerable to a disturbance by exogenous and endogenous factors. We found striking differences in the number of DEGs in both brain structures at both P3 and P10 between OXYS rats and Wistar rats. Most remarkably, in the PFC and hippocampus of OXYS rats at ages P3 and P10, changes in gene expression are widely associated with all key processes involved in AD pathogenesis and which are altered in OXYS rats at different stages of the development of AD signs [20,24,25,26]. Gene expression changes at P3 and P10 are related to specific molecular processes such as neuronal plasticity, lipid metabolic processes, immune processes, cerebrovascular processes, and mitochondrial function.

It is noteworthy that here we detected changes in expression of genes related to Aβ function. The expression profile of genes associated with APP processing is downregulated in the brain of OXYS rats in the early postnatal period. Growing data on APP and Aβ suggests that they perform important physiological functions in the brain; the complete absence of the *App* gene in transgenic mice causes severe neurological deficits [40]. Some studies provide strong evidence that a decrease in Aβ clearance or a decrease in phagocytosis by microglia—rather than an Aβ peptide overproduction by cleavage of the APP—may be involved in AD [41]. In OXYS rats, Aβ accumulates with age, but its level in the brain increases most pronouncedly from the age of 1 year during a decrease in the expression of genes associated with Aβ clearance [20,26] and microglial hypofunction [23,42,43]. Here we noticed that at P3 and P10, DEGs in the PFC and hippocampus are related to Aβ binding and regulation of Aβ formation; at P10, DEGs in both brain regions are related to Aβ clearance. In addition, an interesting and perhaps important result in relation to development of AD pathology is the underexpression of the *Abca7* gene (an important genetic determinant of late-onset AD [27]) in both brain regions of OXYS rats already in the early postnatal period. As results showed, the expression of *Abca7* is decreased in the brain of OXYS rats at P20 and at 5 and 18 months (*p* < 0.05) too. Recently, it was revealed that patients with low levels of ABCA7 develop AD neuropathology at a younger age, those with intermediate ABCA7 amounts develop it later, and the patients who developed it very late have high ABCA7 levels, just as the youngest controls, suggesting that ABCA7 acts as a blocker of AD in the early stage of this disease [44]. Lipid composition of cellular membranes can influence secretase activities and then APP processing and Aβ synthesis; therefore, it can be hypothesized that ABCA7 levels and functioning can directly modulate APP processing and subsequently Aβ production [40].

The major finding in our study is differential expression of more than 10% of genes related to mitochondrial function already at P3 and P10 in the brain of OXYS rats. In terms of the function, among them, there are genes associated primarily with mitochondrial respiratory chain complexes I and IV. Recently, in the hippocampus [25,45] and PFC [26], we found that already at the preclinical stage (age P20), OXYS rats manifest some characteristic ultrastructural changes and decreased activity of respiratory complexes I, IV, and V as well as changes in the expression of genes related to mitochondrial function; these alterations progress with the development of AD-like pathology but without upregulation of ROS production (observed only in 20-day-old OXYS rats). On the other hand, in comparison to complexes I and IV, deficiencies of complex II, III, or V activities in AD are not as pronounced [46]. In this respect, it is perhaps of interest that complex I and IV are partially encoded by mtDNA [47]. It should be emphasized that in the hippocampus at both ages and in the PFC at P10, the expression of genes *Mt-nd1* to *Mt-nd6* was lower in OXYS rats in the current work. Late-onset AD is generally thought to be sporadic epidemiologically, albeit with a genetic factor involved, and in many ways, with unique genetic rules of mtDNA, including heteroplasmy, mitotic segregation, and maternal inheritance [47]. Some have hypothesized that accumulation of somatic mtDNA mutations plays a major part in the development of AD and perhaps even represents a primary cause. It has been reported that brains of AD patients have a high level of a ~5 kbp “common deletion” [48]. As noted previously, in OXYS rats, the level of deleted mtDNA in the hippocampus is higher as compared to Wistar rats, both at the stages preceding the development of AD signs and during their progression [49]. The highest level of the ~5 kbp “common deletion” in OXYS rats is seen at P10 [49]. Summarizing the data from our present work and previous studies, we can conclude that some AD-like-pathology-associated changes may arise during stages where declining brain mitochondrial function could still be accommodated and compensated for or could arise after the declining brain mitochondrial function crossed a critical level at which successful compensation is no longer possible.

Our current analysis of the gene expression changes that occur during normal brain maturation in Wistar rats and the features of brain maturation in OXYS rats by age P10 showed similar pathways that are related to synaptic and immune processes, the cell cycle, focal adhesion, and RNA processes in the PFC and hippocampus. By contrast, functional analysis of DEGs exclusive for each rat strain revealed fundamental differences in the processes and their activity during brain maturation. The main differences are associated with the opposite directions of changes related to mitochondrial function. In Wistar rats, the upregulated exclusive DEGs were found to be related to mitochondrial processes, whereas in OXYS rats, exclusive DEGs were downregulated. In addition, the vascular processes were downregulated in Wistar rats and upregulated in OXYS rats by age P10. Recently, we showed that changes in the expression of the genes functionally associated with cerebrovascular processes are detectable in OXYS rats early in life (already at age P20) [24]. In that report, the major alterations in cerebrovascular processes of OXYS rats proved to be associated with blood vessel development, circulatory system processes, the VEGF signaling pathway, and vascular smooth muscle contraction; at preclinical (age P20) and early stages (5 months) of the AD-like pathology, these processes were upregulated and then downregulated by the age of 18 months. It can be added that the functional analysis of DEGs exclusively downregulated in OXYS rats by age P10 revealed the GO term “telomere maintenance”. Telomere shortening is one of the key hallmarks of aging, and the rate of telomere shortening also correlates with the rate of AD progression [50]. More recently, we demonstrated that leukocyte telomere length is significantly shorter in OXYS rats than in age-matched Wistar rats already at the age of 3 months [51].

Our current comparative gene expression analysis in the PFC and hippocampus of OXYS rats in the period preceding the development of AD signs and during their manifestation and active progression revealed 36 common (among five ages in total) DEGs, and three of them had unidirectional expression changes (upregulation) throughout the lifespan (*Smpd4*, *Thoc3*, and *Exosc8*). Today, the mechanisms that underlie the functions of neuronal and glial cells are unclear, and many studies indicate that sphingolipids are some of the main actors [33]. In the brain, sphingolipids play crucial roles by regulating the rate of growth, differentiation, and death of CNS cells. A disturbance of the balance among different classes of sphingolipids leads to changes in the fate and functions of neuronal cells. SMPD4 is highly expressed during the first 8 weeks after conception in the fetal period and then its expression steadily decreases during postnatal life [52]. In the present work, the highest level of *Smpd4* mRNA in the PFC and hippocampus of Wistar and OXYS rats was noted at P3, and this level decreased with age in rats of both strains, but in OXYS rats this level was higher throughout the entire postnatal development. Sphingolipids are molecules involved in the processing and aggregation of Aβ and in the signal transduction of a cytotoxic signal induced by Aβ. Analysis of genes encoding sphingomyelinases’ expression has revealed their upregulation in the brain of patients with AD [33]. Therefore, sphingomyelinases may be promising targets for drugs preventing neurodegenerative impairments in AD.

Another gene whose expression is increased in the brain of OXYS rats throughout the lifespan is *Thoc3*; the encoded protein is a part of the highly conserved TREX complex, which plays a key role in mRNA biogenesis, in many cellular mechanisms and, as recently shown, in the pathogenesis of cancer and neurodegenerative diseases [34]. TREX is a conserved multisubunit complex essential for embryogenesis, organogenesis, and cellular differentiation throughout life. By linking transcription, mRNA processing, and export together, TREX exerts a physiologically important action in the gene expression pathway. TREX prevents DNA damage and regulates the cell cycle by ensuring optimal gene expression. It must be noted that functions of all the components of this complex have not yet been studied sufficiently well. It is believed that a key step in the understanding of the functions of this complex will be elucidation of its RNA substrates throughout the transcriptome. Complete determination of the composition, function, and interactions of TREX will clarify the molecular basis of a variety of diseases.

The third gene whose expression is upregulated in the brain of OXYS rats from birth is *Exosc8*, which encodes a subunit of exosome RNA, an evolutionarily conserved complex that is crucial for the processing and degradation of various RNAs. It was initially established that exosome RNA genes are essential for viability [35]. For example, *Exosc3* knockout mice are not viable [53]. Quite recently, the first mutation was found in genes of RNA exosome subunits: in *EXOSC3* and a little later in *EXOSC8*. Mutations in these genes are associated with cerebellar hypoplasia: a neurodegenerative disease characterized by psychomotor deficits, hypomyelination, and spinal muscular atrophy from birth; however, amino acid changes in different exosome subunits cause distinct, tissue-specific disease phenotypes. Prior to the discovery of these mutations, it had been assumed that any disturbances of the exosomal RNA complex would have similar functional consequences. Therefore, the biggest surprise is the finding that mutations in these genes give such diverse tissue-specific phenotypes [35].

Furthermore, among DEGs with unidirectional changes in expression throughout the lifespan in OXYS rats, *Ift140*, *Tctn1,* and *Ift81* deserve attention. These genes are related to cilia: the dynamic microtubule-based organelles. The primary cilium is increasingly viewed as a hub for neuronal signaling. Cilia defects underlie a growing list of human disorders with overlapping phenotypes such as developmental delays and cognitive and memory deficits. Consistently with these observations, cilia play an important part in brain development, particularly in neurogenesis and neuronal migration. These findings suggest that at the systems level, a deeper understanding of how ciliary proteins function together may provide new mechanistic insights into the molecular etiologies of nervous-system defects. Recently, evidence emerged indicating a relation between ciliary protein dysfunction and AD [36]. In the hippocampus of OXYS rats, signs of structural disorders of axonal myelin fibers, structural changes in microtubules, and their disorganization [43] are reported to increase with age and may be largely due to hyperphosphorylation of the tau protein [20].

In conclusion, here we performed for the first time a comparative analysis of changes in rat brain transcriptomes from infancy to the advanced stage of AD-like pathology. It is striking that the largest and most comparable differences in gene expression and related processes were observed in the early postnatal period and at the stage of severe stage of the pathology. As our results show, a decrease in the efficiency of the formation of neural networks in the brain of OXYS rats at an early age obviously contributes to the development of AD signs. What causes this phenomenon remains unclear, but characteristic shortening of gestational age, low birth weight, and delayed brain development in infancy—typical of these rats—allow us to regard them as major risk factors of emergence of this disease-like pathology later in life. Further investigation in this field would be warranted, to elucidate the causal relation between delayed brain development in infancy and neurodegeneration.

## 4. Materials and Methods

### 4.1. Animals

The OXYS rat strain was developed at the Institute of Cytology and Genetics (ICG), SB RAS (Novosibirsk, Russia), from a Wistar stock by selection for susceptibility to the cataractogenic effect of a galactose-rich diet and brother-sister mating of highly susceptible rats. At present, we have the 120th generation of OXYS rats. This senescence-accelerated strain and age-matched male Wistar rats (parental strain) were obtained from the Breeding Experimental Animal Laboratory of the ICG SB RAS (Novosibirsk, Russia). The animals were kept under standard laboratory conditions (22 ± 2 °C, 60% relative humidity, and 12 h light/12 h dark cycle) and had ad libitum access to standard rodent feed (PK-120-1, Laboratorsnab, Ltd., Moscow, Russia) and water.

### 4.2. Tissue Preparation

Male pups of OXYS and Wistar strains were decapitated at P3 and P10; the brains were carefully excised, and the PFC and hippocampus were isolated rapidly, placed in RNALater (Ambion, catalog # AM7020), frozen, and stored at −20 °C until analysis. Frozen rat tissues were lysed with the TRIzol Reagent (Invitrogen, cat. # 15596–018), and total RNA was isolated. RNA quality and quantity were evaluated on an Agilent Bioanalyzer (Agilent).

### 4.3. Illumina Sequencing

More than 40 million single-end reads 50 bp long were obtained for each sample of cortical RNA, by Illumina nonstranded sequencing on an Illumina GAIIx instrument at the Genoanalitika Lab, Moscow, Russia [http://www.genoanalytica.ru/, (accessed on 1 September 2021)] in accordance with standard Illumina protocols (mRNA-Seq Sample Prep Kit). Briefly, polyadenylated mRNA was purified from total RNA using Sera-Mag Magnetic Oligo (dT) beads and then broken into small fragments by means of divalent cations and heating. Using a reverse transcriptase and random primers, we synthesized the first- and second-strand cDNAs. The cDNA was processed in an end repair reaction with T4 DNA polymerase and Klenow DNA polymerase to blunt the termini. An “A” base was then added to the 3′ end of the blunt phosphorylated DNA fragments, and an Illumina adaptor with a single T overhang at its 3′ end was then ligated to the end of the DNA fragment, for hybridization in a single-read flow cell. After that, a size range of cDNA templates was selected, and these fragments were amplified on a cluster station using the Single-Read Cluster Generation Kit v2. Sequencing-by-synthesis (SBS) at 50-nucleotide length was performed by means of SBS v4 reagents on a Genome Analyzer IIx running the SCS2.8 software (Illumina).

### 4.4. RNA-Seq Analysis

The RNA-Seq data were obtained as described earlier [24]. Briefly, the sequencing data were preprocessed using the Cutadapt tool (https://cutadapt.readthedocs.io, http://www.genoanalytica.ru/, (accessed on 1 September 2021)) to remove adapters and low-quality sequences. The resulting reads were mapped onto the Rnor_5.0 reference genome assembly in the TopHat2 software (https://ccb.jhu.edu/software/tophat/, (accessed on 1 September 2021)). The data were then converted into gene count tables using by means of ENSEMBL and RefSeq gene annotation data. The resulting tables were subjected to the analysis of differential gene expression in the DESeq2 software (https://bioconductor.org/packages/release/bioc/html/DESeq2.html (accessed on 1 September 2021)). Genes with P_adj_ < 0.05 were designated as differentially expressed.

### 4.5. Comparisons with Previous Rat mRNA Expression Studies

To understand the molecular genetic mechanisms underlying the development, aging, and dementia, we performed a comparative gene expression analysis in the PFC and hippocampus of OXYS rats (the 112th generation) and Wistar rats using current and previous RNA-seq data from 20-day-old and 5- and 18-month-old animals [24,25,26].

### 4.6. Functional Analysis and Construction of Gene Interaction Networks

To identify the Gene Ontology (GO) terms overrepresented in a differentially expressed genes (DEGs) list, the detected DEGs were subjected to functional enrichment analyses by means of the DAVID (http://david.abcc.ncifcrf.gov/summary.jsp; accessed on 1 September 2022) tool. Pathway analysis of the DEGs was conducted in WebGestalt GSAT (http://www.webgestalt.org/option.php; accessed on 1 September 2022) using the KEGG pathways (http://www.genome.jp/kegg/; accessed on 1 September 2022) and WikiPathways (https://www.wikipathways.org; accessed on 1 September 2022). The gene interaction networks were identified on the GeneMANIA web server (http://www.genemania.org/; accessed on 1 September 2022) and by means of STRING (https://string-db.org/; accessed on 1 September 2022).

### 4.7. Analysis of Expression of Genes Related to Aβ Function and Mitochondrial Function

The list of genes related to Aβ function was obtained in the Rat Genome Database (RGD; 118 rat genes, https://rgd.mcw.edu/; accessed on 1 September 2022). The list of genes related to mitochondrial function was compiled by comparing the gene lists in MitoCarta 2.0 (1158 mouse genes, www.broadinstitute.org/pubs/MitoCarta; accessed on 1 September 2022), Integrated Mitochondrial Protein Index, IMPI Q3 2017 database (1483 rat genes, http://impi.mrc-mbu.cam.ac.uk/; accessed on 1 September 2022), and RGD (1232 rat genes, https://rgd.mcw.edu/; accessed on 1 September 2022). The analyses of genes related to mitochondrial respiratory chain complexes I–V and hypoxia and genes involved in oxidative stress and antioxidant defense were performed using the list of genes in PathCards (https://pathcards.genecards.org/; accessed on 1 September 2022), RGD (https://rgd.mcw.edu/; accessed on 1 September 2022), and in ref. [31], respectively.

## Data Availability

Raw data are available from the corresponding author upon request.

## Supplementary Materials

The supporting information can be downloaded at: https://www.mdpi.com/article/10.3390/ijms24021462/s1.
