# Supplementary material for: The Rat Brain Transcriptome: From Infancy to Aging and Sporadic Alzheimer’s Disease-like Pathology
Source: Int J Mol Sci. 2023 Jan 11;24(2):1462. doi: 10.3390/ijms24021462 (PMC9865438; doi:10.3390/ijms24021462)
Supplement: Supplementary file 1 [file ijms-24-01462-s001.zip › ijms-2107643-supplementary Figures.pdf]

Supplementary Figures.

Natalia A. Stefanova and Nataliya G. Kolosova. The Rat Brain Transcriptome: from Infancy to Aging and Sporadic Alzheimer's Disease-Like Pathology

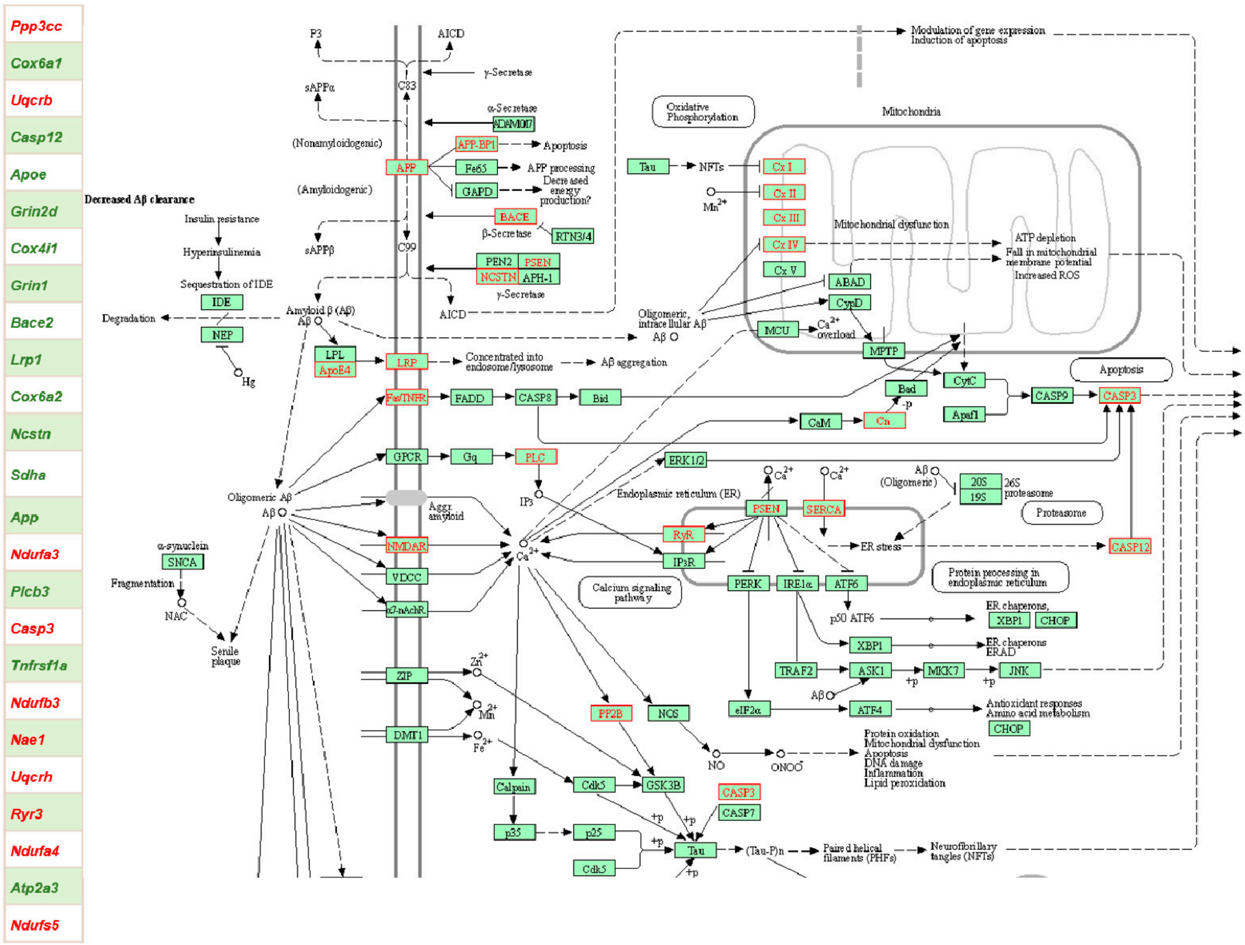

Supplementary Figure S1. According to the KEGG pathway analysis, in the PFC of OXYS rats at age P3, 25 DEGs (of these, 15 were downregulated [green gene symbol], and 10 were upregulated [red gene symbol]) were related to the Alzheimer's disease pathway ($P_{adj} < 0.05$). The genes whose expression is altered in OXYS rats are highlighted in red in the scheme. These genes can be divided into three clusters. The first one is the downregulated genes involved in the processing and degradation of amyloid beta (*Apoe*, *Bace2*, *Lrp*, and *App*). The second one is the genes encoding glutamate receptor (*Grin1* and *Grin2*), and the third one is the genes associated with mitochondrial respiratory chain complexes. In the scheme, the first four complexes are highlighted in red, but the first one is the most affected: the expression of genes encoding subunits of NADH-dehydrogenase is increased (*Ndufa3*, *Ndufb3*, *Ndufa4*, and *Ndufs5*), and in the fourth one, the expression of cytochromes is reduced (*Cox6a1*, *Cox4i1*, and *Cox6a2*).

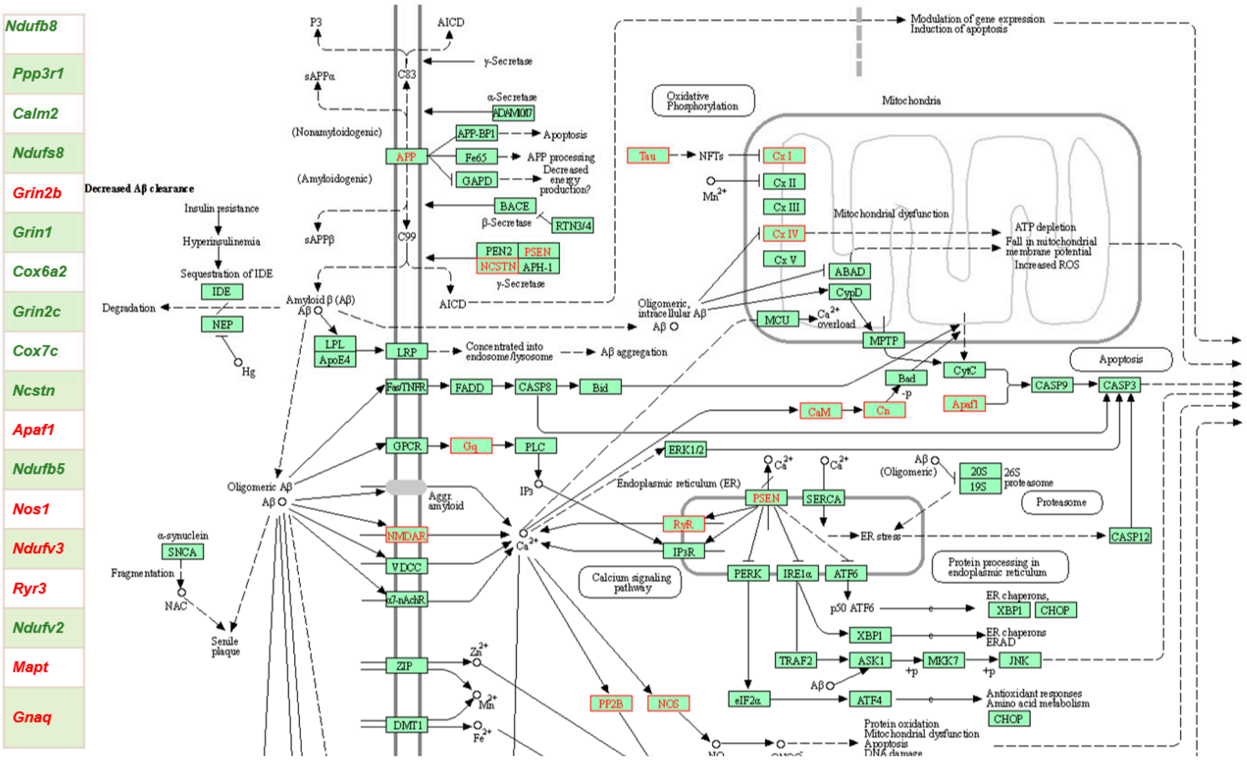

Supplementary Figure S2. According to the KEGG pathway analysis, in the PFC of OXYS rats at age P10, 18 DEGs [of these, 11 were downregulated (green gene symbol), and 7 were upregulated (red gene symbol)] were related to the Alzheimer's disease pathway ($P_{adj} < 0.05$). The genes whose expression is altered in OXYS rats are highlighted in red in the scheme. Among them, the expression of genes related to complexes I and IV of the mitochondrial respiratory chain is reduced (*Ndufb8*, *Ndufs8*, *Ndufb5*, *Ndufv2*, *Cox6a2*, and *Cox7c*). The expression of genes encoding glutamate receptor *Grin1* and *Grin2c* is downregulated, and *Grin2b* is upregulated. The expression of the gene encoding the microtubule-associated protein (*Mapt*) is increased.

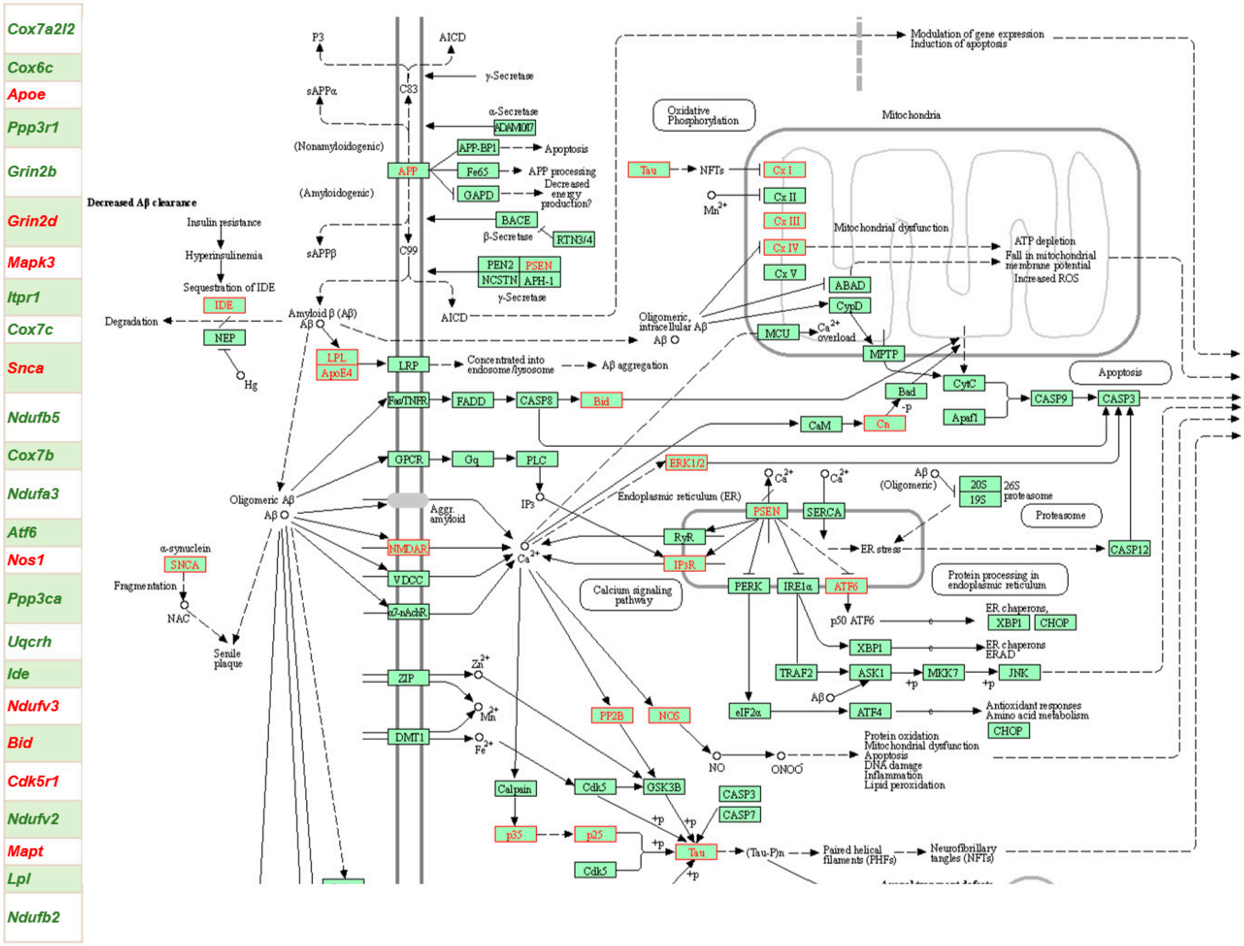

Supplementary Figure S3. According to the KEGG pathway analysis, in the hippocampus of OXYS rats at age P10, 25 DEGs [of these, 16 were downregulated (green gene symbol), and 9 were upregulated (red gene symbol)] were related to the Alzheimer's disease pathway ($P_{adj} < 0.05$). The genes whose expression is altered in OXYS rats are marked red in the scheme. Among them, the expression of genes involved in the processing and degradation of amyloid beta (*Apoe*, *Ide*, and *Lpl*) and of genes of glutamate receptors (*Grin2b* and *Grin2d*) is both reduced and increased. As in the PFC of OXYS rats at P10, the expression of the *Mapt* gene is increased and the expression of genes of the mitochondrial respiratory chain complexes I and IV (*Ndufb5*, *Ndufa3*, *Ndufv3*, *Ndufv2*, *Ndufb2*, *Cox7a2l2*, *Cox6c*, *Cox7c,* and *Cox7b*) is reduced.

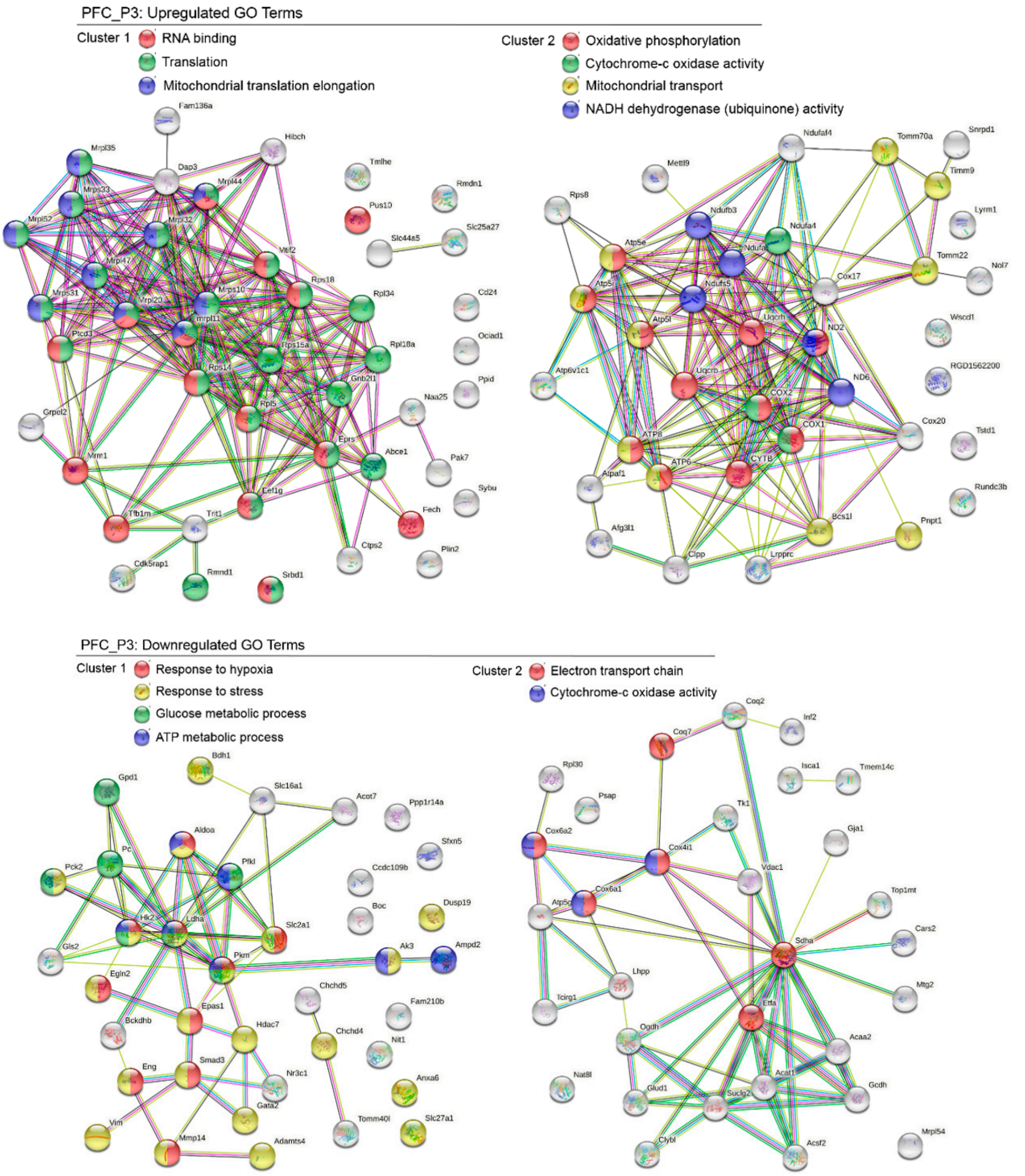

Supplementary Figure S4. Genes differentially expressed between OXYS and Wistar rats and related to the mitochondrial function in the PFC at P3 according to cluster analysis by STRING. Upregulated GO terms: GO terms enriched within a set of upregulated DEGs.

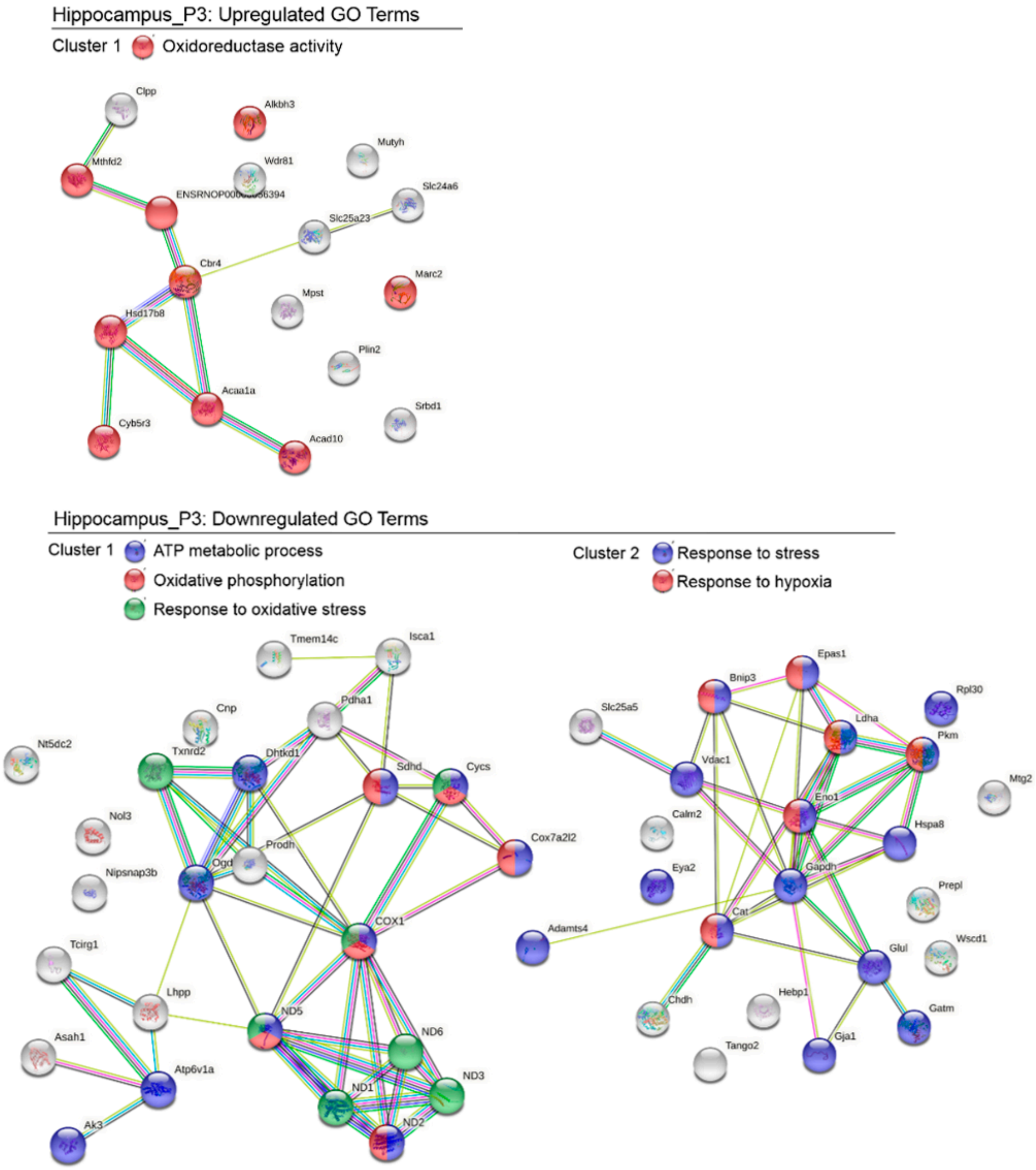

Supplementary Figure S5. Genes differentially expressed between OXYS and Wistar rats and related to the mitochondrial function in the hippocampus at P3 according to cluster analysis by STRING.

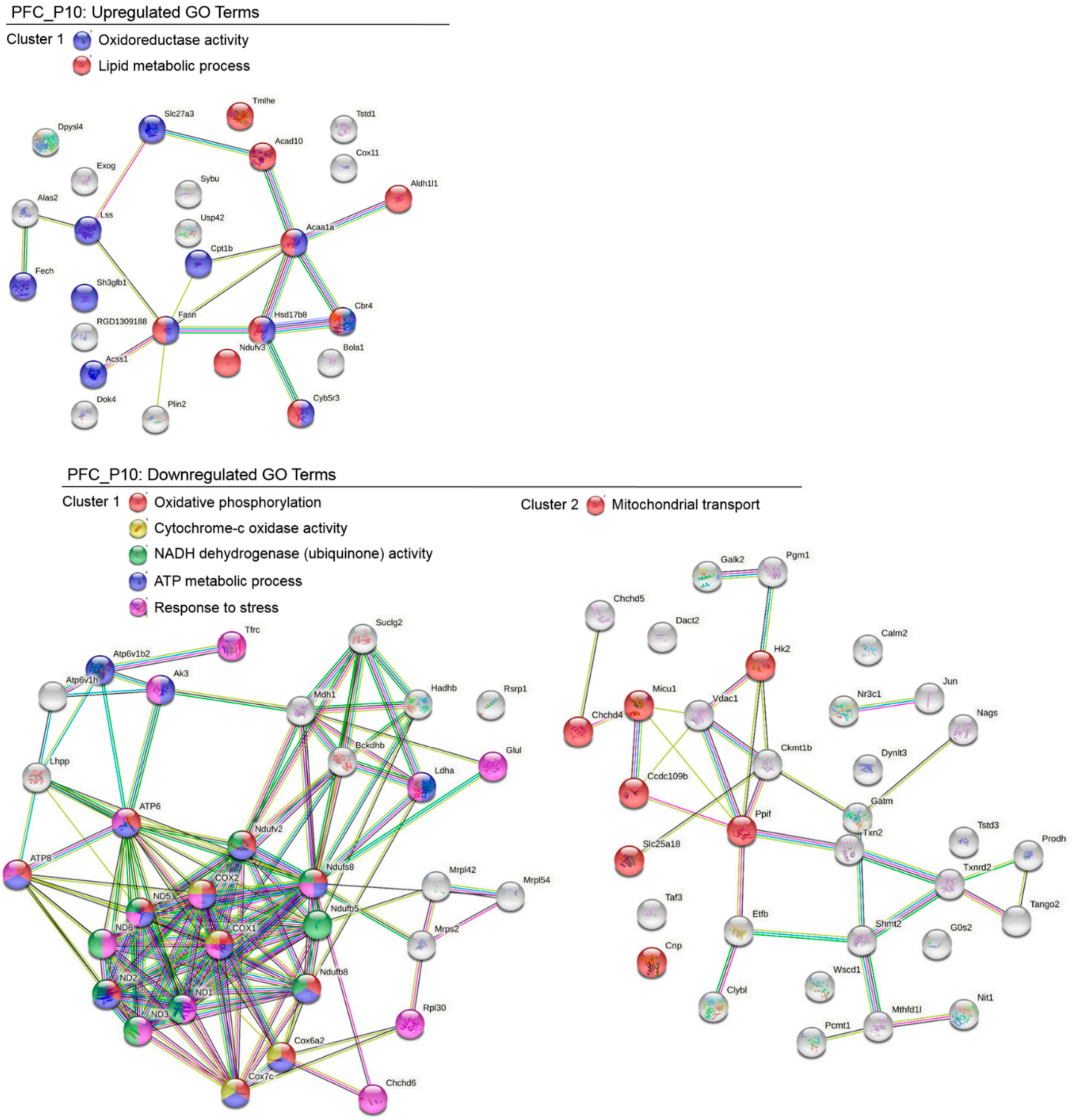

Supplementary Figure S6. Genes differentially expressed between OXYS and Wistar rats and related to the mitochondrial function in the PFC at P10 according to cluster analysis by STRING.

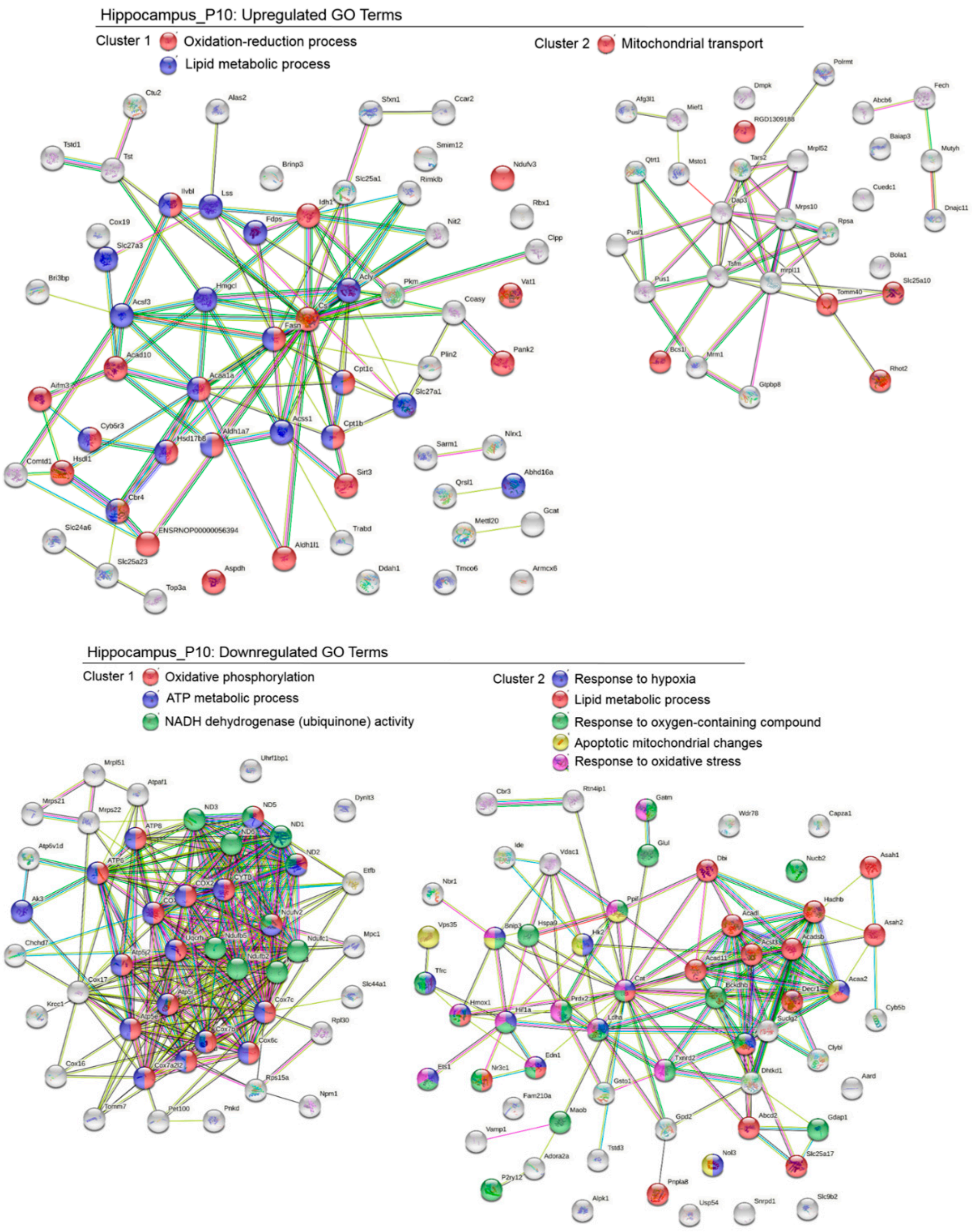

Supplementary Figure S7. Genes differentially expressed between OXYS and Wistar rats and related to the mitochondrial function in the hippocampus at P10 according to cluster analysis by STRING.
