# Supplementary material for: The Rat Brain Transcriptome: From Infancy to Aging and Sporadic Alzheimer’s Disease-like Pathology
Source: Int J Mol Sci. 2023 Jan 11;24(2):1462. doi: 10.3390/ijms24021462 (PMC9865438; doi:10.3390/ijms24021462)
Supplement: Supplementary file 1 [file ijms-24-01462-s001.zip › ijms-2107643-supplementary Tables.pdf]

Supplementary Tables.

Natalia A. Stefanova and Nataliya G. Kolosova. The Rat Brain Transcriptome: from Infancy to Aging and Sporadic Alzheimer's Disease-Like Pathology

Supplementary Table S1. The top 100 DEGs in the PFC of OXYS rats at age P3. FC: fold change.

Gene ID	Gene symbol	Gene name	log_2FC	P_{adj}
**ENSRNOG00000032825**	*LOC100362027*	ribosomal protein L30-like	2.92	7.89E-167
**ENSRNOG00000005975**	*Rpl30*	ribosomal protein L30	-1.86	4.80E-137
**ENSRNOG00000023226**	*S100a10*	S100 calcium binding protein A10	1.28	1.15E-59
**ENSRNOG00000050419**	*Avil*	advillin	-1.84	2.03E-44
**ENSRNOG00000019339**	*Ttll11*	tubulin tyrosine ligase like11	1.74	3.32E-43
**ENSRNOG00000031993**	*Prim1*	DNA primase subunit 1	-0.85	3.41E-41
**ENSRNOG00000016217**	*Gtpbp4*	GTP binding protein 4	1.16	2.05E-30
**ENSRNOG00000005924**	*Dstn*	destrin	0.7	8.65E-30
**ENSRNOG00000024825**	*Fam163a*	family with sequence similarity 163	-1.62	1.40E-29
**ENSRNOG00000020533**	*Htra1*	HtrA serine peptidase 1	-1.39	1.61E-29
**ENSRNOG00000020411**	*Sec23ip*	SEC23 interacting protein	0.53	3.14E-27
**ENSRNOG00000007998**	*Ssb*	small RNA binding exonuclease protection factor La	0.45	1.09E-25
**ENSRNOG00000000104**	*Thoc3*	THO complex 3	0.64	4.33E-25
**ENSRNOG00000007060**	*Plin2*	perilipin 2	0.63	3.42E-23
**ENSRNOG00000016977**	*Calb2*	calbindin 2	0.98	1.13E-22
**ENSRNOG00000011094**	*Efcab6*	EF-hand calcium binding domain 6	1.03	1.80E-22
**ENSRNOG00000048273**	*Apod*	apolipoprotein D	-1.34	3.50E-21
**ENSRNOG00000047052**	*Clpp*	caseinolytic mitochondrial matrix peptidase proteolytic subunit	0.8	2.72E-20
**ENSRNOG00000009037**	*Sulf1*	sulfatase 1	1.3	3.32E-20
**ENSRNOG00000002969**	*Itpkb*	inositol-trisphosphate 3-kinase B	-0.47	1.84E-19
**ENSRNOG00000013766**	*Acaa2*	acetyl-CoA acyltransferase 2	-0.62	1.87E-19
**ENSRNOG00000010189**	*Rps24*	ribosomal protein S24	0.6	1.62E-18
**ENSRNOG00000015020**	*Idh1*	isocitrate dehydrogenase (NADP(+)) 1	0.5	2.03E-18
**ENSRNOG00000011027**	*Uba5*	ubiquitin-like modifier activating enzyme 5	-0.43	5.69E-17
**ENSRNOG00000025121**	*Pla2g3*	phospholipase A2	-1.08	1.98E-16
**ENSRNOG00000010268**	*Vom2r44*	vomeronasal 2 receptor 44	-4.33	2.19E-16
**ENSRNOG00000016545**	*Ift140*	intraflagellar transport 140	-0.59	3.77E-16
**ENSRNOG00000033299**	*Mt-atp8*	mitochondrially encoded ATP synthase 8	0.41	5.66E-16
**ENSRNOG00000008917**	*Ehbp1*	EH domain binding protein 1	0.32	6.47E-15
**ENSRNOG00000029726**	*Gstm1*	glutathione S-transferase mu 1	0.46	8.73E-15
**ENSRNOG00000030700**	*Mt-co3*	mitochondrially encoded cytochrome c oxidase III	0.27	9.03E-15
**ENSRNOG00000033517**	*LOC100360791*	tumor protein	-1.42	2.05E-14
**ENSRNOG00000004777**	*Esf1*	ESF1 nucleolar pre-rRNA processing protein homolog	0.59	2.15E-14
**ENSRNOG00000018400**	*Golm1*	golgi membrane protein 1	0.74	3.82E-14
**ENSRNOG00000029042**	*Mt-nd6*	mitochondrially encoded NADH dehydrogenase 6	0.41	5.80E-14
**ENSRNOG00000001294**	*Ift81*	intraflagellar transport 81	0.55	6.34E-14

**ENSRNOG00000028622**	*Pnpla1*	patatin-like phospholipase domain containing 1	-3.33	1.20E-13
**ENSRNOG00000016346**	*Prkcd*	protein kinase C	-0.8	1.29E-13
**ENSRNOG00000059344**	*Tpcn1*	two pore segment channel 1	-0.47	1.32E-13
**ENSRNOG00000001347**	*Adam1a*	ADAM metallopeptidase domain 1a	0.74	1.44E-13
**ENSRNOG00000040297**	*Ttc37*	tetratricopeptide repeat domain 37	0.63	1.69E-13
**ENSRNOG00000060168**	*Prkx*	protein kinase	0.5	3.11E-13
**ENSRNOG00000014137**	*Fbln1*	fibulin 1	-0.79	3.71E-13
**ENSRNOG00000014702**	*Elovl2*	ELOVL fatty acid elongase 2	-0.34	6.05E-13
**ENSRNOG00000024953**	*RGD1559441*	similar to MIC2L1	-0.61	7.27E-13
**ENSRNOG00000004346**	*Notch3*	notch receptor 3	-0.61	1.25E-12
**ENSRNOG00000023633**	*Crabp1*	cellular retinoic acid binding protein 1	1.92	1.78E-12
**ENSRNOG00000002916**	*Ca4*	carbonic anhydrase 4	-1.47	4.03E-12
**ENSRNOG00000020373**	*Dap3*	death associated protein 3	0.49	4.68E-12
**ENSRNOG00000004616**	*Npm1*	nucleophosmin 1	0.33	5.68E-12
**ENSRNOG00000023521**	*Map3k10*	mitogen activated protein kinase kinase kinase 10	-0.33	8.10E-12
**ENSRNOG00000018434**	*Stab1*	stabilin 1	-1.03	8.60E-12
**ENSRNOG00000027797**	*Nit2*	nitrilase family	0.99	2.80E-11
**ENSRNOG00000020579**	*Col7a1*	collagen type VII alpha 1 chain	-0.9	2.80E-11
**ENSRNOG00000010438**	*Cpt1b*	carnitine palmitoyltransferase 1B	0.62	3.17E-11
**ENSRNOG00000006086**	*Lynx1*	Ly6/neurotoxin 1	-1.09	3.82E-11
**ENSRNOG00000052256**	*Dact3*	dishevelled-binding antagonist of beta-catenin 3	-0.32	3.87E-11
**ENSRNOG00000009531**	*Col9a3*	collagen type IX alpha 3 chain	-0.45	5.88E-11
**ENSRNOG00000012634**	*Fbxo10*	F-box protein 10	-0.51	7.25E-11
**ENSRNOG00000001469**	*Eln*	elastin	-0.81	9.01E-11
**ENSRNOG00000007454**	*Aloxe3*	arachidonate lipoxygenase 3	0.78	1.54E-10
**ENSRNOG00000000454**	*RT1-DOb*	RT1 class II locus DOb	-4.62	1.80E-10
**ENSRNOG00000009205**	*Lmo4*	LIM domain only 4	0.3	2.01E-10
**ENSRNOG00000007657**	*Col27a1*	collagen type XXVII alpha 1 chain	-0.73	2.43E-10
**ENSRNOG00000007564**	*Evc*	EvC ciliary complex subunit 1	-1.08	2.47E-10
**ENSRNOG00000001795**	*Itgb5*	integrin subunit beta 5	-0.53	2.78E-10
**ENSRNOG00000007338**	*Fbln2*	fibulin 2	-0.67	3.02E-10
**ENSRNOG00000011977**	*Sema5a*	semaphorin 5A	0.66	3.17E-10
**ENSRNOG00000010886**	*Nfe2l3*	nuclear factor	0.74	3.54E-10
**ENSRNOG00000001304**	*Bcr*	BCR activator of RhoGEF and GTPase	-0.32	4.44E-10
**ENSRNOG00000025209**	*Plxnd1*	plexin D1	-0.66	4.44E-10
**ENSRNOG00000009892**	*Adamts15*	ADAM metallopeptidase with thrombospondin type 1 motif	-0.67	4.61E-10
**ENSRNOG00000027125**	*Mboat1*	membrane bound O-acyltransferase domain containing 1	-0.83	4.84E-10
**ENSRNOG00000057556**	*Pdzrn3*	PDZ domain containing RING finger 3	-0.41	7.54E-10
**ENSRNOG00000001990**	*Ugt2b10*	UDP glucuronosyltransferase 2 family	3.24	1.14E-09
**ENSRNOG00000000233**	*Grm6*	glutamate metabotropic receptor 6	0.84	1.14E-09
**ENSRNOG00000002871**	*Rbm25l1*	RNA binding motif protein 25-like 1	0.42	1.21E-09
**ENSRNOG00000002618**	*Ivns1abp*	influenza virus NS1A binding protein	0.23	1.38E-09
**ENSRNOG00000009094**	*Nudt4*	nudix hydrolase 4	-0.29	1.43E-09

**ENSRNOG00000017369**	*Mustn1*	musculoskeletal embryonic nuclear protein 1	-0.96	1.56E-09
**ENSRNOG00000012906**	*Bcas1*	breast carcinoma amplified sequence 1	-0.74	1.75E-09
**ENSRNOG00000013954**	*Alpl*	alkaline phosphatase	-0.7	1.90E-09
**ENSRNOG00000007142**	*B3gat1*	beta-1	0.43	1.92E-09
**ENSRNOG00000022784**	*Fzd10*	frizzled class receptor 10	-1.08	3.54E-09
**ENSRNOG00000017945**	*Serf1*	small EDRK-rich factor 1	0.49	3.70E-09
**ENSRNOG00000014795**	*Nr2f1*	nuclear receptor subfamily 2	-0.3	3.76E-09
**ENSRNOG00000004161**	*Mtif2*	mitochondrial translational initiation factor 2	0.6	5.29E-09
**ENSRNOG00000020673**	*Pbxip1*	PBX homeobox interacting protein 1	-0.57	5.36E-09
**ENSRNOG00000000903**	*Asl*	argininosuccinate lyase	-0.49	7.55E-09
**ENSRNOG00000047768**	*Lamb2*	laminin subunit beta 2	-0.6	7.67E-09
**ENSRNOG00000012660**	*Postn*	periostin	0.9	7.86E-09
**ENSRNOG00000004737**	*Cd48*	Cd48 molecule	-0.69	9.08E-09
**ENSRNOG00000012067**	*Fam111a*	family with sequence similarity 111	0.94	1.03E-08
**ENSRNOG00000051688**	*Syt15*	synaptotagmin 15	1.89	1.10E-08
**ENSRNOG00000011910**	*Hnrnpr*	heterogeneous nuclear ribonucleoprotein R	0.44	1.26E-08
**ENSRNOG00000002563**	*Mcts1*	MCTS1	0.37	1.27E-08
**ENSRNOG00000008607**	*U2surp*	U2 snRNP-associated SURP domain containing	0.33	1.29E-08
**ENSRNOG00000001316**	*Anapc5*	anaphase-promoting complex subunit 5	0.21	1.41E-08
**ENSRNOG00000012392**	*Dnajc2*	DnaJ heat shock protein family (Hsp40) member C2	0.36	1.59E-08
**ENSRNOG00000019322**	*Notch1*	notch receptor 1	-0.38	1.77E-08

Supplementary Table S2. The top 100 DEGs in the PFC of OXYS rats at age P10.

Gene ID	Gene symbol	Gene name	log_2FC	P_{adj}
**ENSRNOG00000005975**	*Rpl30*	ribosomal protein L30	-1.94	1.37E-142
**ENSRNOG00000032825**	*LOC100362027*	ribosomal protein L30-like	2.62	6.84E-114
**ENSRNOG00000001963**	*Mx2*	MX dynamin like GTPase 2	-2.96	2.16E-66
**ENSRNOG00000047052**	*Clpp*	caseinolytic mitochondrial matrix peptidase proteolytic subunit	1.08	4.57E-50
**ENSRNOG00000038999**	*RT1-A1*	RT1 class Ia	2.10	1.57E-46
**ENSRNOG00000027797**	*Nit2*	nitrilase family	1.30	1.31E-44
**ENSRNOG00000031993**	*Prim1*	DNA primase subunit 1	-0.94	1.90E-38
**ENSRNOG00000050419**	*Avil*	advillin	-1.9	1.27E-35
**ENSRNOG00000047321**	*Hba-a2*	hemoglobin alpha	1.30	3.28E-35
**ENSRNOG00000029886**	*Hba-a1*	hemoglobin alpha	1.08	3.33E-34
**ENSRNOG00000000104**	*Thoc3*	THO complex 3	0.69	1.47E-32
**ENSRNOG00000012906**	*Bcas1*	breast carcinoma amplified sequence 1	-1.32	3.36E-32
**ENSRNOG00000012067**	*Fam111a*	family with sequence similarity 111	1.34	1.24E-31
**ENSRNOG00000016545**	*Ift140*	intraflagellar transport 140	-0.62	4.29E-31
**ENSRNOG00000020411**	*Sec23ip*	SEC23 interacting protein	0.56	5.69E-31
**ENSRNOG00000017496**	*Cnp*	2'.3'-Cyclic Nucleotide 3' Phosphodiesterase	-0.59	3.79E-30
**ENSRNOG00000045989**	*Hba-a3*	hemoglobin alpha	1.39	1.68E-26
**ENSRNOG00000018606**	*Olr59*	olfactory receptor 59	-1.92	1.52E-25
**ENSRNOG00000023226**	*S100a10*	S100 calcium binding protein A10	1.02	4.95E-23
**ENSRNOG00000031171**	*Wdr46*	WD repeat domain 46	0.53	1.07E-21
**ENSRNOG00000022844**	*Pdzrn4*	PDZ domain containing RING finger 4	1.00	2.63E-21
**ENSRNOG00000052444**	*Samd9*	sterile alpha motif domain containing 9	-0.94	1.07E-20
**ENSRNOG00000033517**	*LOC100360791*	tumor protein	-1.46	5.89E-20
**ENSRNOG00000007060**	*Plin2*	perilipin 2	0.88	1.63E-19
**ENSRNOG00000001316**	*Anapc5*	anaphase-promoting complex subunit 5	0.31	2.75E-19
**ENSRNOG00000025676**	*Gask1a*	golgi associated kinase 1A	1.72	1.25E-18
**ENSRNOG00000033615**	*Mt-nd3*	mitochondrially encoded NADH dehydrogenase 3	-0.42	1.15E-17
**ENSRNOG00000033402**	*Gsta6*	glutathione S-transferase alpha 6	-5.09	5.85E-17
**ENSRNOG00000000167**	*Alas2*	5'-aminolevulinate synthase 2	1.12	7.36E-17
**ENSRNOG00000020923**	*Tuft1*	tuftelin 1	-1.71	1.09E-16
**ENSRNOG00000016977**	*Calb2*	calbindin 2	0.73	1.20E-16
**ENSRNOG00000029778**	*Maob*	monoamine oxidase B	-0.6	2.64E-16
**ENSRNOG00000019339**	*Ttll11*	tubulin tyrosine ligase like11	0.90	3.01E-16
**ENSRNOG00000027940**	*Plppr3*	phospholipid phosphatase related 3	0.37	4.01E-16
**ENSRNOG00000002419**	*Plp1*	proteolipid protein 1	-1.32	1.13E-15
**ENSRNOG00000010268**	*Vom2r44*	vomeronasal 2 receptor 44	-4.06	1.79E-15
**ENSRNOG00000023908**	*Egfem1*	EGF-like and EMI domain containing 1	1.44	3.69E-15
**ENSRNOG00000007456**	*Calb1*	calbindin 1	-0.56	6.08E-15

**ENSRNOG00000009740**	*Slco1c1*	solute carrier organic anion transporter family	-0.34	1.38E-14
**ENSRNOG00000049560**	*Glul*	glutamate-ammonia ligase	-0.47	1.80E-14
**ENSRNOG00000001123**	*Spring1*	RIKEN cDNA 2410131K14 gene	0.50	2.38E-14
**ENSRNOG00000005248**	*Slc1a4*	solute carrier family 1 member 4	0.34	2.80E-14
**ENSRNOG00000017689**	*Itih3*	inter-alpha trypsin inhibitor	0.52	3.48E-14
**ENSRNOG00000030712**	*RT1-A2*	RT1 class Ia	-3.38	6.26E-14
**ENSRNOG00000017369**	*Mustn1*	musculoskeletal	-0.93	8.32E-14
**ENSRNOG00000001347**	*Adam1a*	ADAM metallopeptidase domain 1a	0.86	1.56E-13
**ENSRNOG00000007597**	*Rhpn1*	rhophilin	1.00	1.87E-13
**ENSRNOG00000048273**	*Apod*	apolipoprotein D	-1.15	3.58E-13
**ENSRNOG00000040297**	*Ttc37*	tetratricopeptide repeat domain 37	0.56	6.30E-13
**ENSRNOG00000018343**	*Isca1*	iron-sulfur cluster assembly 1	-0.35	7.02E-13
**ENSRNOG00000000981**	*Scarb1*	scavenger receptor class B	0.36	7.32E-13
**ENSRNOG00000014866**	*Pign*	phosphatidylinositol glycan anchor biosynthesis	1.23	1.85E-12
**ENSRNOG00000005924**	*Dstn*	destrin	0.43	3.13E-12
**ENSRNOG00000020951**	*Slc4a1*	solute carrier family 4 member 1	1.30	4.51E-12
**ENSRNOG00000020533**	*Htra1*	HtrA serine peptidase 1	-1.03	4.63E-12
**ENSRNOG00000010438**	*Cpt1b*	carnitine palmitoyltransferase 1B	0.77	4.87E-12
**ENSRNOG00000052873**	*Npnt*	nephronectin	0.50	5.04E-12
**ENSRNOG00000047505**	*Tubb4a*	tubulin	-0.41	6.46E-12
**ENSRNOG00000015020**	*Idh1*	isocitrate dehydrogenase (NADP(+)) 1	0.56	6.50E-12
**ENSRNOG00000050378**	*Pcdha4*	protocadherin alpha 4	1.01	1.35E-11
**ENSRNOG00000013654**	*Cbln2*	cerebellin 2 precursor	-0.76	1.40E-11
**ENSRNOG00000011027**	*Uba5*	ubiquitin-like modifier activating enzyme 5	-0.38	1.46E-11
**ENSRNOG00000033261**	*Fam107a*	family with sequence similarity 107	-0.52	1.47E-11
**ENSRNOG00000016753**	*Slc14a1*	solute carrier family 14 member 1 (Kidd blood group)	-0.64	1.63E-11
**ENSRNOG00000001294**	*Ift81*	intraflagellar transport 81	0.50	4.43E-11
**ENSRNOG00000019424**	*Aspdh*	aspartate dehydrogenase domain containing	0.82	4.54E-11
**ENSRNOG00000009629**	*Car2*	carbonic anhydrase 2	-0.64	6.51E-11
**ENSRNOG00000023233**	*LOC102546648*	uncharacterized LOC102546648	-0.72	7.10E-11
**ENSRNOG00000008934**	*Tmem65*	transmembrane protein 65	0.47	8.24E-11
**ENSRNOG00000024402**	*Focad*	focadhesin	-0.44	8.98E-11
**ENSRNOG00000054955**	*Adgra1*	adhesion G protein-coupled receptor A1	-0.75	1.03E-10
**ENSRNOG00000028622**	*Pnpla1*	patatin-like phospholipase domain containing 1	-1.53	1.51E-10
**ENSRNOG00000000454**	*RT1-DOb*	RT1 class II	-1.11	1.60E-10
**ENSRNOG00000014508**	*Mgll*	monoglyceride lipase	-0.42	2.24E-10
**ENSRNOG00000018991**	*Gsn*	gelsolin	0.71	2.77E-10
**ENSRNOG00000009054**	*Elmod1*	ELMO domain containing 1	-0.32	3.56E-10
**ENSRNOG00000003984**	*Apln*	apelin	-0.68	3.98E-10
**ENSRNOG00000014331**	*Zfp810*	zinc finger protein 810	0.34	4.34E-10
**ENSRNOG00000024689**	*Hopx*	HOP homeobox	-0.78	4.67E-10

**ENSRNOG00000033791**	*Apc2*	APC regulator of WNT signaling pathway 2	0.27	6.71E-10
**ENSRNOG00000003881**	*Nit1*	nitrilase 1	-0.5	9.99E-10
**ENSRNOG00000004737**	*Cd48*	Cd48 molecule	-0.67	1.76E-09
**ENSRNOG00000002916**	*Ca4*	carbonic anhydrase 4	-0.79	1.82E-09
**ENSRNOG00000033220**	*Oas1f*	2 ' -5 ' oligoadenylate synthetase 1F	1.39	2.15E-09
**ENSRNOG00000022957**	*Ctxn3*	cortexin 3	-1.31	2.46E-09
**ENSRNOG00000016217**	*Gtpbp4*	GTP binding protein 4	0.66	2.80E-09
**ENSRNOG00000009094**	*Nudt4*	nudix hydrolase 4	-0.34	3.08E-09
**ENSRNOG00000013766**	*Acaa2*	acetyl-CoA acyltransferase 2	-0.66	3.20E-09
**ENSRNOG00000018524**	*Ezr*	ezrin	-0.35	3.87E-09
**ENSRNOG00000014996**	*Katna1*	katanin catalytic subunit A1	-0.71	5.67E-09
**ENSRNOG00000004781**	*Crmp1*	collapsin response mediator protein 1	0.28	6.24E-09
**ENSRNOG00000005697**	*Slc6a11*	solute carrier family 6 member 11	0.25	8.96E-09
**ENSRNOG00000019519**	*Idnk*	Idnk	0.98	9.49E-09
**ENSRNOG00000029386**	*RT1-N2*	RT1 class Ib	-1.19	1.06E-08
**ENSRNOG00000000809**	*Atat1*	alpha tubulin acetyltransferase 1	0.25	1.06E-08
**ENSRNOG00000020623**	*Aldh16a1*	aldehyde dehydrogenase 16 family	0.52	1.06E-08
**ENSRNOG00000028708**	*Ntsr1*	neurotensin receptor 1	0.48	1.33E-08
**ENSRNOG00000008970**	*Pcdh17*	protocadherin 17	0.34	1.43E-08
**ENSRNOG00000016456**	*Il33*	interleukin 33	-0.67	1.63E-08
**ENSRNOG00000032813**	*Mdc1*	mediator of DNA damage checkpoint 1	0.46	1.87E-08

Supplementary Table S3. The top 100 DEGs in the hippocampus of OXYS rats at age P3.

Gene ID	Gene symbol	Gene name	log_2FC	P_{adj}
**ENSRNOG00000018991**	*Gsn*	gelsolin	2.54	0.00E+00
**ENSRNOG00000005975**	*Rpl30*	ribosomal protein L30	-2.41	4.51E-131
**ENSRNOG00000023226**	*S100a10*	S100 calcium binding protein A10	1.38	2.06E-84
**ENSRNOG00000031993**	*Prim1*	DNA primase subunit 1	-0.99	5.29E-62
**ENSRNOG00000032825**	*LOC100362027*	ribosomal protein L30-like	3.60	5.52E-46
**ENSRNOG00000019339**	*Ttll11*	tubulin tyrosine ligase like11	1.64	1.90E-39
**ENSRNOG00000031171**	*Wdr46*	WD repeat domain 46	0.67	1.31E-33
**ENSRNOG00000000104**	*Thoc3*	THO complex 3	0.68	6.72E-31
**ENSRNOG00000013766**	*Acaa2*	acetyl-CoA acyltransferase 2	-0.82	8.74E-30
**ENSRNOG00000025676**	*Gask1a*	golgi associated kinase 1A	2.08	1.99E-28
**ENSRNOG00000017689**	*Itih3*	inter-alpha trypsin inhibitor, heavy chain 3	1.06	1.47E-24
**ENSRNOG00000002969**	*Itpkb*	inositol-trisphosphate 3-kinase B	-0.57	6.40E-23
**ENSRNOG00000000436**	*Egfl8*	EGF-like-domain, multiple 8	1.26	1.07E-21
**ENSRNOG00000007060**	*Plin2*	perilipin 2	0.66	1.56E-21
**ENSRNOG00000001316**	*Anapc5*	anaphase-promoting complex subunit 5	0.28	1.23E-20
**ENSRNOG00000012906**	*Bcas1*	breast carcinoma amplified sequence 1	-0.64	2.28E-19
**ENSRNOG00000010438**	*Cpt1b*	carnitine palmitoyltransferase 1B	0.66	2.84E-18
**ENSRNOG00000004430**	*Cep131*	centrosomal protein 131	0.48	3.58E-18
**ENSRNOG00000012562**	*Grin3b*	glutamate ionotropic receptor NMDA type subunit 3B	0.80	7.64E-18
**ENSRNOG00000032063**	*Gfral*	GDNF family receptor alpha like	1.99	1.04E-17
**ENSRNOG00000016545**	*Ift140*	intraflagellar transport 140	-0.55	1.27E-17
**ENSRNOG00000021183**	*Rcor2l1*	REST corepressor 2-like 1	-3.02	1.94E-17
**ENSRNOG00000006545**	*Septin7*	septin 7	-0.44	2.60E-17
**ENSRNOG00000001963**	*Mx2*	MX dynamin like GTPase 2	-2.07	1.66E-16
**ENSRNOG00000019629**	*Lamp1*	lysosomal-associated membrane protein 1	0.27	1.66E-16
**ENSRNOG00000018343**	*Isca1*	iron-sulfur cluster assembly 1	-0.35	5.69E-16
**ENSRNOG00000017105**	*Dpyd*	dihydropyrimidine dehydrogenase	-1.1	1.98E-15
**ENSRNOG00000015225**	*Gramd2b*	GRAM domain containing 2B	-0.72	2.88E-15
**ENSRNOG00000014702**	*Elovl2*	ELOVL fatty acid elongase 2	-0.44	2.88E-15
**ENSRNOG00000028622**	*Pnpla1*	patatin-like phospholipase domain containing 1	-3.22	3.34E-15
**ENSRNOG00000001347**	*Adam1a*	ADAM metallopeptidase domain 1a	0.77	3.34E-15
**ENSRNOG00000033615**	*Mt-nd3*	mitochondrially encoded NADH dehydrogenase 3	-0.48	4.09E-15
**ENSRNOG00000020411**	*Sec23ip*	SEC23 interacting protein	0.44	4.50E-15
**ENSRNOG00000029386**	*RT1-N2*	RT1 class Ib, locus N2	-2.12	6.97E-15
**ENSRNOG00000011027**	*Uba5*	ubiquitin-like modifier activating enzyme 5	-0.45	7.78E-15
**ENSRNOG00000016346**	*Prkcd*	protein kinase C, delta	-0.72	9.05E-15
**ENSRNOG00000017496**	*Cnp*	2',3'-cyclic nucleotide 3' phosphodiesterase	-0.44	2.06E-14
**ENSRNOG00000007939**	*Naprt*	nicotinate phosphoribosyltransferase	0.89	2.30E-14

**ENSRNOG00000013228**	*Scrg1*	stimulator of chondrogenesis 1	-0.53	2.42E-14
**ENSRNOG00000020533**	*Htra1*	HtrA serine peptidase 1	-0.91	4.08E-14
**ENSRNOG00000020923**	*Tuft1*	tuftelin 1	-1.26	1.17E-13
**ENSRNOG00000040297**	*Ttc37*	tetratricopeptide repeat domain 37	0.53	1.33E-13
**ENSRNOG00000019424**	*Aspdh*	aspartate dehydrogenase domain containing	0.78	2.48E-13
**ENSRNOG00000009033**	*Cntn2*	contactin 2	0.48	5.28E-13
**ENSRNOG00000018400**	*Golm1*	golgi membrane protein 1	0.66	6.38E-13
**ENSRNOG00000016217**	*Gtpbp4*	GTP binding protein 4	0.71	6.48E-13
**ENSRNOG00000007869**	*Wscd1*	WSC domain containing 1	-0.36	1.41E-12
**ENSRNOG00000004554**	*Dcn*	decorin	-1.3	1.46E-12
**ENSRNOG00000001990**	*Ugt2b10*	UDP glucuronosyltransferase 2 family, polypeptide B10	2.85	1.61E-12
**ENSRNOG00000000454**	*RT1-DOb*	RT1 class II, locus DOb	-5.16	1.79E-12
**ENSRNOG00000003881**	*Nit1*	nitrilase 1	-0.46	1.84E-12
**ENSRNOG00000004793**	*Ncln*	nicalin	0.31	3.53E-12
**ENSRNOG00000010268**	*Vom2r44*	vomeronasal 2 receptor 44	-4.39	3.70E-12
**ENSRNOG00000047052**	*Clpp*	caseinolytic mitochondrial matrix peptidase proteolytic subunit	0.54	4.44E-12
**ENSRNOG00000002538**	*Epb41l5*	erythrocyte membrane protein band 4,1 like 5	-1.25	7.03E-12
**ENSRNOG00000009047**	*Sln*	sarcolipin	-2.79	7.39E-12
**ENSRNOG00000009636**	*Scrn1*	secernin 1	0.24	8.79E-12
**ENSRNOG00000016995**	*Zap70*	zeta chain of T cell receptor associated protein kinase 70	-0.78	1.06E-11
**ENSRNOG00000038074**	*LOC498555*	similar to 60S acidic ribosomal protein P2	-8.04	1.14E-11
**ENSRNOG00000043451**	*Spp1*	secreted phosphoprotein 1	-2.29	1.45E-11
**ENSRNOG00000002419**	*Plp1*	proteolipid protein 1	-0.65	1.05E-10
**ENSRNOG00000004737**	*Cd48*	Cd48 molecule	-0.58	1.66E-10
**ENSRNOG00000020373**	*Dap3*	death associated protein 3	0.38	1.73E-10
**ENSRNOG00000015020**	*Idh1*	isocitrate dehydrogenase (NADP(+)) 1	0.34	3.04E-10
**ENSRNOG00000007564**	*Evc*	EvC ciliary complex subunit 1	-0.74	4.80E-10
**ENSRNOG00000025581**	*Vwa2*	von Willebrand factor A domain containing 2	0.71	5.68E-10
**ENSRNOG00000006375**	*Vdac1*	voltage-dependent anion channel 1	-0.29	9.35E-10
**ENSRNOG00000019294**	*Stk16*	serine/threonine kinase 16	0.34	1.46E-09
**ENSRNOG00000033220**	*Oas1f*	2 ' -5 ' oligoadenylate synthetase 1F	1.82	1.46E-09
**ENSRNOG00000002705**	*Vps4b*	vacuolar protein sorting 4 homolog B	0.36	1.60E-09
**ENSRNOG00000015347**	*Trim45*	tripartite motif-containing 45	1.06	1.76E-09
**ENSRNOG00000000728**	*Clic2*	chloride intracellular channel 2	-2.05	2.05E-09
**ENSRNOG00000018977**	*Ap3d1*	adaptor related protein complex 3 subunit delta 1	0.30	2.38E-09
**ENSRNOG00000003984**	*Apln*	apelin	-0.68	2.97E-09
**ENSRNOG00000000522**	*Cpne5*	copine 5	-0.42	3.27E-09
**ENSRNOG00000016516**	*Mbp*	myelin basic protein	-0.66	4.32E-09
**ENSRNOG00000016371**	*Slc18b1*	solute carrier family 18 member B1	-0.56	4.78E-09
**ENSRNOG00000026109**	*Prmt1*	protein arginine methyltransferase 1	-0.33	5.75E-09
**ENSRNOG00000033402**	*Gsta6*	glutathione S-transferase alpha 6	-4.4	1.04E-08
**ENSRNOG00000020363**	*Med25*	mediator complex subunit 25	0.24	1.06E-08

**ENSRNOG00000014075**	*Clybl*	citrate lyase beta like	-0.9	1.18E-08
**ENSRNOG00000019519**	*Idnk*	Idnk, gluconokinase	0.91	1.20E-08
**ENSRNOG00000023633**	*Crabp1*	cellular retinoic acid binding protein 1	1.48	1.33E-08
**ENSRNOG00000039024**	*Spice1*	spindle and centriole associated protein 1	0.47	1.38E-08
**ENSRNOG00000026226**	*Hook1*	hook microtubule-tethering protein 1	-0.48	1.67E-08
**ENSRNOG00000013171**	*Grm2*	glutamate metabotropic receptor 2	-0.78	2.30E-08
**ENSRNOG00000025042**	*Pde3a*	phosphodiesterase 3A	0.68	3.03E-08
**ENSRNOG00000017897**	*Adam8*	ADAM metallopeptidase domain 8	0.89	3.39E-08
**ENSRNOG00000032042**	*Zfta*	similar to RIKEN cDNA 2700081O15	0.26	3.89E-08
**ENSRNOG00000007324**	*Plxna2*	plexin A2	0.28	5.19E-08
**ENSRNOG00000020634**	*Pih1d1*	PIH1 domain containing 1	-0.57	6.62E-08
**ENSRNOG00000011745**	*Psma1*	proteasome 20S subunit alpha 1	-0.27	7.64E-08
**ENSRNOG00000006867**	*Etv1*	ETS variant transcription factor 1	-0.24	9.23E-08
**ENSRNOG00000060168**	*Prkx*	protein kinase, X-linked	0.39	1.07E-07
**ENSRNOG00000026049**	*Qrsl1*	glutaminyl-tRNA amidotransferase subunit QRSL1	0.64	1.07E-07
**ENSRNOG00000042432**	*Nek11*	NIMA-related kinase 11	-1.24	1.17E-07
**ENSRNOG00000001294**	*Ift81*	intraflagellar transport 81	0.34	1.60E-07
**ENSRNOG00000048152**	*Myo1b*	myosin Ib	0.39	1.60E-07
**ENSRNOG00000000470**	*Vps52*	VPS52 subunit of GARP complex	0.38	1.61E-07
**ENSRNOG00000038999**	*RT1-A1*	RT1 class Ia, locus A1	1.22	1.66E-07

Supplementary Table S4. The top 100 DEGs in the hippocampus of OXYS rats at age P10.

Gene ID	Gene symbol	Gene name	log_2FC	P_{adj}
**ENSRNOG00000018991**	*Gsn*	gelsolin	2.57	5.38E-208
**ENSRNOG00000005975**	*Rpl30*	ribosomal protein L30	-2.18	1.47E-143
**ENSRNOG00000032825**	*LOC100362027*	ribosomal protein L30-like	2.70	3.53E-122
**ENSRNOG00000032902**	*Ybx1-ps3*	Y box protein 1 related, pseudogene 3	2.55	6.11E-73
**ENSRNOG00000029042**	*Mt-nd6*	mitochondrially encoded NADH dehydrogenase 6	-0.62	2.91E-62
**ENSRNOG00000031993**	*Prim1*	DNA primase subunit 1	-1.06	9.47E-56
**ENSRNOG00000001963**	*Mx2*	MX dynamin like GTPase 2	-2.78	1.41E-49
**ENSRNOG00000047052**	*Clpp*	caseinolytic mitochondrial matrix peptidase proteolytic subunit	0.87	1.62E-38
**ENSRNOG00000025676**	*Gask1a*	golgi associated kinase 1A	1.57	1.24E-29
**ENSRNOG00000033615**	*Mt-nd3*	mitochondrially encoded NADH dehydrogenase 3	-0.64	9.01E-29
**ENSRNOG00000000104**	*Thoc3*	THO complex 3	0.74	2.21E-27
**ENSRNOG00000007604**	*Igsf8*	immunoglobulin superfamily, member 8	0.43	4.93E-25
**ENSRNOG00000023633**	*Crabp1*	cellular retinoic acid binding protein 1	1.21	2.68E-24
**ENSRNOG00000026589**	*Dpy19l1*	dpy-19 like C-mannosyltransferase 1	0.61	7.86E-24
**ENSRNOG00000058006**	*Sncg*	synuclein, gamma	1.65	7.96E-24
**ENSRNOG00000007060**	*Plin2*	perilipin 2	0.76	8.68E-23
**ENSRNOG00000031171**	*Wdr46*	WD repeat domain 46	0.67	4.79E-22
**ENSRNOG00000033299**	*Mt-atp8*	mitochondrially encoded ATP synthase 8	-0.43	1.22E-21
**ENSRNOG00000005248**	*Slc1a4*	solute carrier family 1 member 4	0.49	2.70E-21
**ENSRNOG00000005861**	*Hsd11b1*	hydroxysteroid 11-beta dehydrogenase 1	-0.54	1.10E-20
**ENSRNOG00000017689**	*Itih3*	inter-alpha trypsin inhibitor, heavy chain 3	0.97	1.23E-20
**ENSRNOG00000001347**	*Adam1a*	ADAM metallopeptidase domain 1a	1.03	1.49E-20
**ENSRNOG00000016545**	*Ift140*	intraflagellar transport 140	-0.59	2.88E-20
**ENSRNOG00000029386**	*RT1-N2*	RT1 class Ib, locus N2	-1.78	1.43E-19
**ENSRNOG00000050419**	*Avil*	advillin	-1.35	2.53E-19
**ENSRNOG00000013766**	*Acaa2*	acetyl-CoA acyltransferase 2	-0.71	4.80E-19
**ENSRNOG00000003881**	*Nit1*	nitrilase 1	-0.57	6.28E-19
**ENSRNOG00000000436**	*Egfl8*	EGF-like-domain, multiple 8	1.31	8.61E-19
**ENSRNOG00000018343**	*Isca1*	iron-sulfur cluster assembly 1	-0.33	2.08E-18
**ENSRNOG00000010438**	*Cpt1b*	carnitine palmitoyltransferase 1B	0.99	7.80E-18
**ENSRNOG00000023226**	*S100a10*	S100 calcium binding protein A10	0.75	1.48E-17
**ENSRNOG00000021156**	*Vegfb*	vascular endothelial growth factor B	0.55	7.68E-17
**ENSRNOG00000017496**	*Cnp*	2',3'-cyclic nucleotide 3' phosphodiesterase	-0.63	1.12E-16
**ENSRNOG00000030644**	*Mt-nd1*	mitochondrially encoded NADH dehydrogenase 1	-0.33	1.12E-16
**ENSRNOG00000047300**	*Bdkrb2*	bradykinin receptor B2	-2.26	1.68E-16
**ENSRNOG00000012067**	*Fam111a*	family with sequence similarity 111, member A	0.82	2.41E-16
**ENSRNOG00000010268**	*Vom2r44*	vomeronasal 2 receptor 44	-4.06	2.86E-16

**ENSRNOG00000047505**	*Tubb4a*	tubulin, beta 4A class IVa	-0.36	3.91E-16
**ENSRNOG00000027940**	*Plppr3*	phospholipid phosphatase related 3	0.54	5.24E-16
**ENSRNOG00000019629**	*Lamp1*	lysosomal-associated membrane protein 1	0.31	2.35E-15
**ENSRNOG00000031979**	*Mt-atp6*	mitochondrially encoded ATP synthase 6	-0.24	4.96E-15
**ENSRNOG00000012562**	*Grin3b*	glutamate ionotropic receptor NMDA type subunit 3B	1.21	1.21E-14
**ENSRNOG00000001300**	*P2rx4*	purinergic receptor P2X 4	0.74	1.37E-14
**ENSRNOG00000000839**	*Nfkbil1*	NFKB inhibitor like 1	0.72	1.42E-14
**ENSRNOG00000003349**	*Col23a1*	collagen alpha-1(XXIII) chain	1.21	2.48E-14
**ENSRNOG00000004757**	*Tmem158*	transmembrane protein 158	0.48	2.75E-14
**ENSRNOG00000019424**	*Aspdh*	aspartate dehydrogenase domain containing	0.93	2.80E-14
**ENSRNOG00000028185**	*Tstd3*	thiosulfate sulfurtransferase like domain containing 3	-0.58	3.54E-14
**ENSRNOG00000029971**	*Mt-nd5*	mitochondrially encoded NADH dehydrogenase 5	-0.31	8.62E-14
**ENSRNOG00000000321**	*Cd24*	CD24 molecule	0.39	1.49E-13
**ENSRNOG00000019339**	*Ttll11*	tubulin tyrosine ligase like11	0.86	2.52E-13
**ENSRNOG00000000105**	*Cplx2*	complexin 2	-0.25	2.77E-13
**ENSRNOG00000014751**	*Ret*	ret proto-oncogene	0.64	3.44E-13
**ENSRNOG00000030371**	*Mt-co2*	mitochondrially encoded cytochrome c oxidase II	-0.25	5.69E-13
**ENSRNOG00000042985**	*Slc9b1*	solute carrier family 9 member B1	-2.68	6.54E-13
**ENSRNOG00000027125**	*Mboat1*	membrane bound O-acyltransferase domain containing 1	-1.13	6.64E-13
**ENSRNOG00000014310**	*Tmtc4*	transmembrane O-mannosyltransferase targeting cadherins 4	0.46	8.20E-13
**ENSRNOG00000028523**	*Tctn1*	tectonic family member 1	0.59	1.01E-12
**ENSRNOG00000004737**	*Cd48*	Cd48 molecule	-0.69	1.13E-12
**ENSRNOG00000009694**	*Bmp4*	bone morphogenetic protein 4	-0.86	1.25E-12
**ENSRNOG00000015020**	*Idh1*	isocitrate dehydrogenase (NADP(+)) 1	0.48	2.29E-12
**ENSRNOG00000007597**	*Rhpn1*	rhophilin, Rho GTPase binding protein 1	0.80	3.63E-12
**ENSRNOG00000029886**	*Hba-a1*	hemoglobin alpha, adult chain 1	0.65	4.10E-12
**ENSRNOG00000009660**	*Enpp6*	ectonucleotide pyrophosphatase/phosphodiesterase 6	-1.66	6.54E-12
**ENSRNOG00000020489**	*Tmem145*	transmembrane protein 145	0.34	6.54E-12
**ENSRNOG00000007564**	*Evc*	EvC ciliary complex subunit 1	-0.88	1.09E-11
**ENSRNOG00000004793**	*Ncln*	nicalin	0.35	1.14E-11
**ENSRNOG00000007490**	*Gabrr2*	gamma-aminobutyric acid type A receptor rho 2 subunit	-1.38	1.19E-11
**ENSRNOG00000007939**	*Naprt*	nicotinate phosphoribosyltransferase	0.65	1.43E-11
**ENSRNOG00000023851**	*Igsf3*	immunoglobulin superfamily, member 3	0.32	1.60E-11
**ENSRNOG00000020411**	*Sec23ip*	SEC23 interacting protein	0.47	1.60E-11
**ENSRNOG00000005256**	*Zc3h15*	zinc finger CCCH-type containing 15	-0.34	1.64E-11
**ENSRNOG00000012906**	*Bcas1*	breast carcinoma amplified sequence 1	-0.62	2.08E-11
**ENSRNOG00000025745**	*Gpr17*	G protein-coupled receptor 17	-0.42	2.16E-11
**ENSRNOG00000032639**	*Foxo6*	forkhead box O6	0.42	2.48E-11
**ENSRNOG00000012420**	*Bcl9l*	BCL9 like	0.30	2.52E-11
**ENSRNOG00000002916**	*Ca4*	carbonic anhydrase 4	-0.57	3.36E-11

**ENSRNOG00000030712**	*RT1-A2*	RT1 class Ia, locus A2	-3.01	4.80E-11
**ENSRNOG00000014816**	*Slc1a1*	solute carrier family 1 member 1	-0.27	5.20E-11
**ENSRNOG00000047321**	*Hba-a2*	hemoglobin alpha, adult chain 2	0.86	5.20E-11
**ENSRNOG00000016640**	*Dner*	delta/notch-like EGF repeat containing	0.26	5.93E-11
**ENSRNOG00000003463**	*Srebf1*	sterol regulatory element binding transcription factor 1	0.47	7.55E-11
**ENSRNOG00000031033**	*Mt-nd2*	mitochondrially encoded NADH dehydrogenase 2	-0.37	1.47E-10
**ENSRNOG00000001316**	*Anapc5*	anaphase-promoting complex subunit 5	0.29	1.47E-10
**ENSRNOG00000000454**	*RT1-DOb*	RT1 class II, locus DOb	-3.5	1.55E-10
**ENSRNOG00000048273**	*Apod*	apolipoprotein D	-0.82	2.30E-10
**ENSRNOG00000004430**	*Cep131*	centrosomal protein 131	0.43	2.30E-10
**ENSRNOG00000018293**	*Strip1*	striatin interacting protein 1	0.27	3.05E-10
**ENSRNOG00000014605**	*Lig4*	DNA ligase 4	-0.85	3.61E-10
**ENSRNOG00000001516**	*Rapgef4*	Rap guanine nucleotide exchange factor 4	-0.37	3.61E-10
**ENSRNOG00000020363**	*Med25*	mediator complex subunit 25	0.32	3.61E-10
**ENSRNOG00000060407**	*Cacna1i*	calcium voltage-gated channel subunit alpha1 I	0.32	3.70E-10
**ENSRNOG00000051688**	*Syt15*	synaptotagmin 15	2.76	5.14E-10
**ENSRNOG00000020373**	*Dap3*	death associated protein 3	0.40	6.28E-10
**ENSRNOG00000027797**	*Nit2*	nitrilase family, member 2	1.22	6.69E-10
**ENSRNOG00000011946**	*Ptn*	pleiotrophin	-0.29	6.94E-10
**ENSRNOG00000013228**	*Scrg1*	stimulator of chondrogenesis 1	-0.43	7.07E-10
**ENSRNOG00000012060**	*Gucy1b1*	guanylate cyclase 1 soluble subunit beta 1	-0.41	8.41E-10
**ENSRNOG00000033261**	*Fam107a*	family with sequence similarity 107, member A	-0.33	8.41E-10
**ENSRNOG00000029191**	*LOC685067*	similar to guanylate binding protein family, member 6	0.90	9.17E-10

Supplementary Table S5. The most significant clusters (DAVID Enrichment Score, ES > 1.3) of enriched Gene Ontology (GO) terms [biological processes (BP), molecular functions (MF), and cellular components (CC)] for the top 100 DEGs in the PFC and hippocampus of OXYS rats at ages P3 and P10. [1]Each row corresponds to a cluster of Functional Annotation Tool (FAT) GO categories. [2]One to three GO terms showing a significant-enrichment P value in each cluster are listed, indicated by "~".

PFC			Hippocampus		
The age of P3					
GO cluster[1]	Terms[2]	ES	GO cluster[1]	Terms[2]	ES
BP	~extracellular matrix organization	2.75	BP	~intraciliary transport<br>~cilium assembly	1.76
The age of P10					
BP<br>MF	~ oxygen transport<br>~oxygen binding<br>~ heme binding	2.05	CC<br><br><br>MF<br><br><br>BP	~mitochondrial respiratory chain complex I<br>~NADH dehydrogenase (ubiquinone) activity<br>~mitochondrial electron transport, NADH to ubiquinone	3.53
CC	~ focal adhesion<br>~actin cytoskeleton	1.6	CC<br><br>BP	~endoplasmic reticulum<br>~response to drug	2.46
BP	~antigen processing and presentation	1.4	CC	~integral component of membrane	1.51

Supplementary Table S6. Downregulated biological processes common between ages P3 and P10 for OXYS rats according to the STRING database.

GO term	P value	No. of genes	Gene symbol
Common for hippocampus			
Synapse	6.20E-03	18	*Cplx2, Ppp1r2, Htr5b, Vamp4, Tuba1a, Chn2, Glrb, Myo6, Sparcl1, Bnip3, Rpl17, Clcn3, Hapln1, Rpl38, Plekha5, Gopc, Dgkb, Mctp2*
Common for PFC			
Anatomical structure development	2.26E-02	39	*Rph3a, Bdh1, Igfbp7, Olig1, Pik3r6, Chrdl1, Sdc2, Ncstn, Mfng, Tagln2, Hcn2, Oaf, Ckb, Cxcl14, Myo1e, Pcdh8, Rnd1, Efhd2, Scg2, Coq7, Mustn1, Abhd2, Egr3, Akt2, Bcan, Chtf18, Inpp5j, Coro1a, Sptbn2, Ptprn, Apba3, Dbp, Grin1, Fzd2, Bcl2l11, Vgf, Mmd2, Fstl4, Tnfaip3*
Phosphoprotein	3.60E-03	29	*Abhd5, Slc7a1, Rph3a, Bdh1, Sdc2, Tagln2, Hcn2, Ckb, Rrad, Myo1e, Pcdh8, Efhd2, Scg2, Mustn1, Kcnt1, Akt2, Bcan, Habp4, Tef, Inpp5j, Coro1a, Sptbn2, Ptprn, Apba3, Dbp, Grin1, Bcl2l11, Vgf, Sept8*
Common for PFC and hippocampus			
Antigen processing and presentation	2.20E-03	8	*RT1-DOb, RT1-M3-1, RT1-S3, RT1-CE7, RT1-N1, RT1-A2, Slc11a1, Fcgr2a*
Mitochondrion	8.87e-05	16	*Gatm, Txnrd2, Coq2, Nit1, Vdac1, Bckdhb, Coq3, Sox10, Clybl, Tmem14c, Ak3, Isca1, Acad11, Acaa2, Suclg2, Glul*

Supplementary Table S7. Clustering analysis (Top 5) based on the DAVID database. Analysis of the DEGs associated with mitochondrial function in the PFC and hippocampus of OXYS rats at P3 and P10 (Enrichment Score > 1.3).

**PFC, P3**			
Annotation Cluster 1	Enrichment Score: 10.14		
Category	Term	P value	Genes
KEGG_PATHWAY	~oxidative phosphorylation	7.01E-18	*Mt-nd6, Uqcrb, Mt-co1, Cox4i1, Ndufb3, Cox17, Tcirg1, Cox6a1, Mt-nd2, Cox6a2, Uqcrh, Atp5mc1, Mt-atp6, Atp5mg, Atp6v1c1, Atp5me, Lhpp, Ndufa4, Ndufa3, Sdha, Atp5f1e, Mt-atp8, Ndufs5, Mt-co2, Mt-co3*
Annotation Cluster 2	Enrichment Score: 4.65		
GOTERM_CC	~mitochondrial respiratory chain complex IV	6.03E-07	*Mt-co1, Ndufa4, Cox4i1, Mt-co2, Mt-co3, Cox6a1, Cox6a2*
Annotation Cluster 3	Enrichment Score: 4.03		
GOTERM_BP	~response to xenobiotic stimulus	2.46E-05	*Maob, Fech, Arg1, Txnrd2, Txnrd1, Stat3, Dnmt3a, Abat, Sod2, Coq7, Acacb, Sod1, Ldha, Bdh1, Adora2a, Cat, Cd38, Tspo, Plin2, Eng*
Annotation Cluster 4	Enrichment Score: 3.84		
GOTERM_CC	~mitochondrial proton-transporting ATP synthase complex	2.01E-06	*Mt-atp6, Atp5mg, Mt-atp8, Atp5f1e, Atp5me, Atp5mc1*
Annotation Cluster 5	Enrichment Score: 3.80		
UP_KW_BIOLOGICAL_PROCESS	~electron transport	5.63E-09	*Mt-nd6, Cyb5a, Uqcrb, Mt-co1, Ndufa3, Ndufb3, Etfa, Sdha, Mt-nd2, Uqcrh, Ndufs5, Mt-co2, Fads1*
GOTERM_BP	~mitochondrial respiratory chain complex I assembly	1.19E-04	*Mt-nd6, Ndufa3, Ndufs5, Ndufaf4, Ndufb3, Mt-nd2, Bcs1l*
**Hippocampus, P3**			
Annotation Cluster 1	Enrichment Score: 4.96		
UP_KW_BIOLOGICAL_PROCESS	~lipid metabolism	4.97E-06	*Asah1, Acaa2, Ggps1, Bckdhb, Acsl4, Acaa1a, Cpt1c, Hsd17b8, Lss, Acadsb, Cpt1b, Acat1, Cyb5r3, Pdss2, Acad11, Acss1, Slc27a2, Cbr4*
Annotation Cluster 2	Enrichment Score: 4.89		
KEGG_PATHWAY	~oxidative phosphorylation	5.89E-10	*Atp6v1a, Mt-nd6, Mt-nd4l, Mt-nd5, Cox7a2l2, Sdhd, Tcirg1, Mt-nd2, Mt-nd3, Cox6b1, Mt-nd1, Cycs, Ndufv3, Lhpp*
GOTERM_CC	~mitochondrial respiratory chain complex I	3.29E-06	*Mt-nd6, Mt-nd4l, Mt-nd5, Ndufv3, Mt-nd2, Mt-nd3, Mt-nd1*
Annotation Cluster 3	Enrichment Score: 4.82		
GOTERM_BP	~tricarboxylic acid cycle	1.55E-06	*Pdha1, Idh1, Ogdh, Suclg2, Sdhd, Dhtkd1*
Annotation Cluster 4	Enrichment Score: 2.93		
GOTERM_MF	~acyl-CoA dehydrogenase activity	1.95E-03	*Acad11, Acad10, Acadsb*
Annotation Cluster 5	Enrichment Score: 2.88		
KEGG_PATHWAY	~glycolysis / gluconeogenesis	4.47E-04	*Ldha, Pdha1, Pkm, Eno1, Acss1, Gapdh*

**PFC, P10**			
Annotation Cluster 1	Enrichment Score: 9.62		
KEGG_PATHWAY	~oxidative phosphorylation	6.04E-18	*Mt-nd6, Mt-nd4l, Ndufb8, Mt-nd5, Ndufb5, Mt-co1, Atp5f1c, Mt-nd2, Cox7c, Mt-nd3, Cox6a2, Mt-atp8, Mt-nd1, Mt-atp6, Ndufs8, Atp6v1b2, Atp6v1h, Cox11, Mt-co2, Ndufv3, Ndufv2, Lhpp*
Annotation Cluster 2	Enrichment Score: 6.79		
UP_KW_BIOLOGICAL_PROCESS	~lipid metabolism	9.36E-06	*Acaa2, Asah2, Bckdhb, Acsl6, Acaa1a, Hsd17b8, Lss, Cpt1b, Acat1, Hadhb, Cyb5r3, Scp2, Bdh1, Fasn, Mt-co2, Acad11, Acss1, Slc27a3, Cbr4, Fads1*
Annotation Cluster 3	Enrichment Score: 4.93		
GOTERM_BP	~fatty acid beta-oxidation	9.49E-08	*Hadhb, Abcd2, Acaa2, Scp2, Auh, Acaa1a, Cpt1b, Acat1*
GOTERM_MF	~acetyl-CoA C-acetyltransferase activity	1.68E-05	*Hadhb, Acaa2, Acaa1a, Acat1*
Annotation Cluster 4	Enrichment Score: 3.31		
UP_SEQ_FEATURE	~mitochondrial intermembrane	2.70E-08	*Tomm70, Coq2, Maob, Opa1, Mt-co1, Mt-co2, Micu1, Cox7c, Cpt1b, Cox6a2*
GOTERM_CC	~mitochondrial respiratory chain complex IV	1.13E-03	*Mt-co1, Mt-co2, Cox7c, Cox6a2*
Annotation Cluster 5	Enrichment Score: 2.59		
GOTERM_CC_	~peroxisome	4.16E-03	*Abcd2, Prdx5, Scp2, Idh1, Acaa1a, Acad11*
**Hippocampus, P10**			
Annotation Cluster 1	Enrichment Score: 13.13		
KEGG_PATHWAY	~oxidative phosphorylation	4.99E-22	*Mt-nd6, Mt-nd4l, Cox7b, Mt-nd5, Mt-co1, Ndufb5, Cox17, Cox7a2l2, Ndufb2, Mt-nd2, Mt-nd3, Cox7c, Uqcrh, Mt-nd1, Mt-atp6, Ndufv3, Ndufv2, Atp6v1d, Atp5mf, Atp5me, Atp5pd, Ndufa3, Ndufc1, Atp5f1c, Cox6c, Atp5f1e, Mt-atp8, Mt-co2, Mt-co3*
Annotation Cluster 2	Enrichment Score: 5.70		
UP_KW_BIOLOGICAL_PROCESS	~lipid metabolism	3.57E-08	*Asah1, Slc27a1, Acaa2, Asah2, Acaa1a, Hsd17b8, Hmgcl, Cyb5r3, Abhd16a, Acadl, Acad11, Acss1, Cbr4, Decr1, Fdps, Ggps1, Pnpla8, Bckdhb, Acsl6, Acsl3, Cpt1c, Lss, Acadsb, Acsf3, Cpt1b, Hadhb, Acly, Ilvbl, Fasn, Mt-co2, Slc27a3*
Annotation Cluster 3	Enrichment Score: 5.03		
GOTERM_CC	~mitochondrial respiratory chain complex IV	6.65E-07	*Cox7b, Mt-co1, Cox7a2l2, Mt-co2, Mt-co3, Cox6c, Cox7c*
Annotation Cluster 4	Enrichment Score: 4.12		
GOTERM_MF	~proton-transporting ATP synthase activity, rotational mechanism	3.99E-08	*Mt-atp6, Atp5pd, Atp5f1c, Atp5mf, Mt-atp8, Atp5f1e, Atp5me*
Annotation Cluster 5	Enrichment Score: 3.72		
GOTERM_CC	~peroxisome	4.25E-05	*Hmgcl, Fdps, Abcd2, Slc25a17, Pnpla8, Idh1, Cat, Ide, Acaa1a, Acad11*

Supplementary Table S8. Gene pathway analysis based on the KEGG database. The top 50 from 181 significant pathways ($p < 0.01$) that undergo expression changes in the PFC of OXYS rats from age P3 to age P10.

Pathway Name	Overlap/all genes	P_{adj}
Metabolic pathways	609/1169	2.62e-146
Pathways in cancer	202/319	8.00e-67
MAPK signaling pathway	162/269	2.72e-49
Focal adhesion	127/186	1.66e-47
Calcium signaling pathway	125/185	4.49e-46
Regulation of actin cytoskeleton	128/208	1.36e-40
Spliceosome	97/135	1.67e-39
Axon guidance	91/126	2.06e-37
Endocytosis	129/230	4.43e-35
RNA transport	101/156	5.14e-35
Wnt signaling pathway	98/152	8.60e-34
Neurotrophin signaling pathway	87/129	2.72e-32
Insulin signaling pathway	87/131	1.51e-31
ErbB signaling pathway	65/85	1.93e-29
Purine metabolism	93/165	9.23e-26
Fc gamma R-mediated phagocytosis	64/91	1.53e-25
Lysosome	77/124	3.31e-25
Vascular smooth muscle contraction	75/120	7.59e-25
Chemokine signaling pathway	96/178	1.06e-24
Cell cycle	76/124	1.97e-24
Neuroactive ligand-receptor interaction	128/282	1.42e-23
Chronic myeloid leukemia	54/73	1.78e-23
Renal cell carcinoma	52/69	2.75e-23
Oocyte meiosis	71/115	3.32e-23
Osteoclast differentiation	71/119	5.78e-22
Progesterone-mediated oocyte maturation	58/86	6.26e-22
Leukocyte transendothelial migration	69/114	6.44e-22
Ribosome	72/122	6.44e-22
Gap junction	59/89	9.25e-22
Glioma	47/62	2.17e-21
Bacterial invasion of epithelial cells	51/71	2.24e-21
Melanogenesis	62/98	2.90e-21
Phosphatidylinositol signaling system	53/76	3.03e-21
Ubiquitin mediated proteolysis	75/133	3.24e-21
GnRH signaling pathway	62/99	5.79e-21
Long-term potentiation	49/69	2.85e-20
Gastric acid secretion	51/74	3.76e-20
Protein processing in endoplasmic reticulum	84/164	4.85e-20
Proteasome	39/49	2.72e-19

Dilated cardiomyopathy	55/88	1.13e-18
Salivary secretion	51/78	1.23e-18
Adherens junction	48/71	1.64e-18
Natural killer cell mediated cytotoxicity	59/101	5.80e-18
Chagas disease (American trypanosomiasis)	59/101	5.80e-18
Pancreatic cancer	46/68	8.14e-18
Toxoplasmosis	69/130	8.95e-18
Amoebiasis	58/99	8.95e-18
Endometrial cancer	38/50	1.17e-17
Fc epsilon RI signaling pathway	49/76	1.25e-17
Tight junction	69/131	1.45e-17

Supplementary Table S9. Gene pathway analysis based on the KEGG database. The top 50 from 180 significant pathways ($p < 0.01$) that undergo expression changes in the PFC of Wistar rats from age P3 to age P10.

Pathway Name	Overlap/all genes	P_{adj}
Metabolic pathways	590/1169	3.93e-149
Pathways in cancer	180/319	9.18e-54
Calcium signaling pathway	117/185	7.06e-42
MAPK signaling pathway	146/269	4.98e-41
Spliceosome	94/135	4.30e-39
Endocytosis	126/230	4.19e-36
Axon guidance	87/126	7.31e-36
Focal adhesion	109/186	5.56e-35
Regulation of actin cytoskeleton	115/208	1.26e-33
RNA transport	96/156	2.24e-33
Wnt signaling pathway	89/152	1.13e-28
Cell cycle	77/124	2.96e-27
Neuroactive ligand-receptor interaction	129/282	4.32e-27
Fc gamma R-mediated phagocytosis	63/91	2.32e-26
Insulin signaling pathway	78/131	5.80e-26
Oocyte meiosis	71/115	4.72e-25
Progesterone-mediated oocyte maturation	58/86	1.87e-23
Neurotrophin signaling pathway	74/129	2.53e-23
Ubiquitin mediated proteolysis	75/133	5.10e-23
Lysosome	70/124	1.42e-21
Osteoclast differentiation	68/119	2.12e-21
Ribosome	69/122	2.24e-21
ErbB signaling pathway	55/85	4.55e-21
Purine metabolism	82/165	1.95e-20
Chemokine signaling pathway	86/178	2.21e-20
Protein processing in endoplasmic reticulum	81/164	5.20e-20
Phosphatidylinositol signaling system	50/76	9.71e-20
Vascular smooth muscle contraction	66/120	1.11e-19
Huntington's disease	96/218	3.79e-19
Alzheimer's disease	94/212	5.00e-19
Tight junction	68/131	1.82e-18
GnRH signaling pathway	57/99	2.06e-18
Gap junction	53/89	4.31e-18
Leukocyte transendothelial migration	61/114	1.59e-17
Phagosome	83/185	2.50e-17
mRNA surveillance pathway	52/89	2.72e-17
Melanogenesis	55/98	3.70e-17
Fc epsilon RI signaling pathway	47/76	4.09e-17
Gastric acid secretion	46/74	6.62e-17

Renal cell carcinoma	44/69	7.71e-17
Long-term potentiation	44/69	7.71e-17
Inositol phosphate metabolism	40/59	8.60e-17
Toxoplasmosis	65/130	9.70e-17
Glioma	41/62	1.29e-16
Chronic myeloid leukemia	45/73	2.06e-16
T cell receptor signaling pathway	57/109	5.98e-16
Ribosome biogenesis in eukaryotes	48/84	1.35e-15
Pancreatic cancer	42/68	1.93e-15
Long-term depression	43/71	2.39e-15
Chagas disease (American trypanosomiasis)	53/101	5.25e-15

Supplementary Table S10. Gene pathway analysis based on the KEGG database. The top 50 from 167 significant pathways ($p < 0.01$) that undergo expression changes in the hippocampus of OXYS rats from age P3 to age P10.

Pathway Name	Overlap/all genes	P_{adj}
Metabolic pathways	563/1169	3.72e-122
Pathways in cancer	185/319	6.71e-55
Spliceosome	100/135	1.13e-43
MAPK signaling pathway	150/269	8.56e-42
Calcium signaling pathway	118/185	4.28e-41
Focal adhesion	115/186	3.57e-38
Regulation of actin cytoskeleton	121/208	2.05e-36
RNA transport	99/156	2.24e-34
Endocytosis	126/230	2.38e-34
Wnt signaling pathway	95/152	2.70e-32
Cell cycle	82/124	1.60e-30
Oocyte meiosis	78/115	3.09e-30
Neuroactive ligand-receptor interaction	137/282	3.42e-30
Axon guidance	81/126	5.87e-29
Neurotrophin signaling pathway	81/129	6.16e-28
Purine metabolism	93/165	6.73e-27
Phosphatidylinositol signaling system	57/76	6.23e-26
Protein processing in endoplasmic reticulum	91/164	1.08e-25
Fc gamma R-mediated phagocytosis	62/91	1.93e-24
Long-term potentiation	52/69	6.36e-24
Progesterone-mediated oocyte maturation	59/86	1.45e-23
Ribosome	73/122	1.63e-23
Insulin signaling pathway	76/131	2.74e-23
ErbB signaling pathway	58/85	4.81e-23
Ubiquitin mediated proteolysis	76/133	9.42e-23
Gap junction	59/89	1.82e-22
Melanogenesis	62/98	5.71e-22
Gastric acid secretion	52/74	1.05e-21
Osteoclast differentiation	69/119	2.84e-21
Pyrimidine metabolism	60/97	1.36e-20
Dilated cardiomyopathy	56/88	3.88e-20
Bacterial invasion of epithelial cells	49/71	5.30e-20
mRNA surveillance pathway	56/89	8.11e-20
Lysosome	68/124	3.31e-19
GnRH signaling pathway	59/99	3.52e-19
Arrhythmogenic right ventricular cardiomyopathy (ARVC)	48/71	4.58e-19
Tight junction	70/131	6.07e-19
Vascular smooth muscle contraction	66/120	8.39e-19

Long-term depression	47/71	3.85e-18
Glioma	43/62	7.54e-18
Huntington's disease	95/218	1.91e-17
Leukocyte transendothelial migration	62/114	2.01e-17
Prostate cancer	52/87	3.75e-17
Renal cell carcinoma	45/69	4.55e-17
Endometrial cancer	37/50	5.09e-17
Chemokine signaling pathway	82/178	5.72e-17
Alzheimer's disease	92/212	7.74e-17
Chronic myeloid leukemia	46/73	1.36e-16
Adherens junction	45/71	2.12e-16
Hypertrophic cardiomyopathy (HCM)	48/80	4.71e-16

Supplementary Table S11. Gene pathway analysis based on the KEGG database. The top 50 from 177 significant pathways ($p < 0.01$) that undergo expression changes in the hippocampus of Wistar rats from age P3 to age P10.

Pathway Name	Overlap/all genes	P_{adj}
Metabolic pathways	589/1169	5.31e-133
Pathways in cancer	207/319	1.90e-71
Focal adhesion	125/186	1.53e-45
MAPK signaling pathway	157/269	1.75e-45
Endocytosis	136/230	2.41e-40
Cell cycle	91/124	2.67e-38
Spliceosome	95/135	1.64e-37
Calcium signaling pathway	115/185	2.82e-37
Regulation of actin cytoskeleton	123/208	9.94e-37
Neurotrophin signaling pathway	90/129	3.27e-35
Fc gamma R-mediated phagocytosis	71/91	4.24e-33
Ubiquitin mediated proteolysis	89/133	1.09e-32
RNA transport	98/156	1.54e-32
Wnt signaling pathway	96/152	3.44e-32
Progesterone-mediated oocyte maturation	67/86	2.65e-31
Axon guidance	84/126	8.34e-31
Insulin signaling pathway	85/131	6.96e-30
Pancreatic cancer	56/68	1.29e-28
Osteoclast differentiation	78/119	6.02e-28
Oocyte meiosis	76/115	1.28e-27
Phosphatidylinositol signaling system	59/76	1.37e-27
ErbB signaling pathway	63/85	1.89e-27
Neuroactive ligand-receptor interaction	134/282	3.51e-27
Protein processing in endoplasmic reticulum	94/164	4.60e-27
Chronic myeloid leukemia	57/73	5.59e-27
Acute myeloid leukemia	47/55	1.23e-25
Purine metabolism	91/165	1.36e-24
Endometrial cancer	43/50	9.44e-24
Chemokine signaling pathway	94/178	1.52e-23
Gastric acid secretion	54/74	3.58e-23
Ribosome	73/122	8.11e-23
B cell receptor signaling pathway	54/75	9.81e-23
Prostate cancer	59/87	1.22e-22
Bacterial invasion of epithelial cells	52/71	1.64e-22
Renal cell carcinoma	51/69	2.08e-22
Fc epsilon RI signaling pathway	54/76	2.46e-22
Huntington's disease	105/218	3.38e-22
Gap junction	59/89	6.39e-22
Glioma	47/62	1.53e-21

Adherens junction	51/71	1.59e-21
Colorectal cancer	50/69	2.09e-21
T cell receptor signaling pathway	66/109	3.16e-21
GnRH signaling pathway	62/99	4.25e-21
Tight junction	73/131	2.04e-20
Non-small cell lung cancer	41/52	4.57e-20
Lysosome	70/124	4.61e-20
Alzheimer's disease	99/212	7.55e-20
Arrhythmogenic right ventricular cardiomyopathy (ARVC)	49/71	1.41e-19
Long-term potentiation	48/69	1.96e-19
Small cell lung cancer	54/84	2.62e-19
